# Heteronuclear Polarization Transfer under Steady-State Conditions: The INEPT-SSFP Experiment

**DOI:** 10.1021/acs.jpclett.4c02016

**Published:** 2024-10-16

**Authors:** Rihards Aleksis, Elton T. Montrazi, Lucio Frydman

**Affiliations:** Department of Chemical and Biological Physics, Weizmann Institute, 7610001 Rehovot, Israel

## Abstract

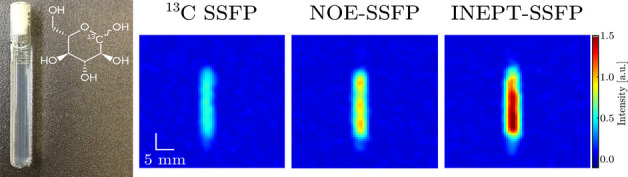

NMR finds a wide range of applications, ranging from fundamental chemistry to medical imaging. The technique, however, has an inherently low signal-to-noise ratio (SNR)—particularly when dealing with nuclei having low natural abundances and/or low γs. In these cases, sensitivity is often enhanced by methods that, similar to INEPT, transfer polarization from neighboring ^1^Hs via *J*-couplings. In 1958, Carr proposed an alternative approach to increase NMR sensitivity, which involves generating and continuously detecting a steady-state transverse magnetization, by applying a train of pulses on an ensemble of noninteracting spins. This study broadens Carr’s steady-state free precession (SSFP) framework to encompass the possibility of adding onto it coherent polarization transfers, allowing one to combine the SNR-enhancing benefits of both INEPT and SSFP into a single experiment. Herein, the derivation of the ensuing INEPT-SSFP (ISSFP) sequences is reported. Their use in ^13^C NMR and MRI experiments leads to ca. 300% improvements in SNR/  over conventional *J*-driven polarization transfer experiments, and sensitivity gains of over 50% over ^13^C SSFP performed in combination with ^1^H decoupling and NOE. These enhancements match well with numerical simulations and analytical evaluations. The conditions needed to optimize these new methods in both spectroscopic and imaging studies are discussed; we also examine their limitations, and the valuable vistas that, in both analytical and molecular imaging NMR, could be opened by this development.

Nuclear amgnetic resonance (NMR) is a primary tool for elucidating the identity and structure of organic and inorganic species,^1,2^ a leading method for characterizing the dynamics and three-dimensional structure of biomacromolecules,^3,4^ and is uniquely capable of detecting and monitoring diseases via imaging (MRI) and spectroscopic imaging (MRSI).^5,6^ Despite this outstanding portfolio, NMR exhibits a notoriously poor sensitivity, particularly when targeting low-γ and/or low-abundance nuclides such as ^13^C or ^15^N. A widespread strategy for boosting the sensitivity of such unreceptive nuclei consists of transferring, via *J*-couplings, polarization from the abundant, high-γ ^1^Hs that typically surround these targets. In solution NMR, such polarization transfers usually proceed via insensitive nuclei enhanced by polarization transfer (INEPT),^7,8^ which is a sequence based on few but carefully timed, phased and calibrated radio-frequency (RF) pulses acting on the targeted nuclei. Early decades of magnetic resonance witnessed an alternative route for enhancing NMR’s signal-to-noise ratio per square-root unit time (SNR_t_), with the introduction of the steady-state free precession (SSFP) sequence (Scheme 1a).^9^ SSFP consists of a train of equidistant RF pulses with flip angles θ and phases ϕ, spaced by repetition time τ_R_ that includes the data acquisition period. As a result of the rapid pulsing, the experiment provides, in the τ_R_ ≪ *T*_2_, *T*_1_ regime, NMR signals that maximize SNR_t_, reaching a steady-state emission equivalent to half the total equilibrium magnetization *M*_0_, when *T*_1_ = *T*_2_. Although short repetition times τ_R_ ≪ *T*_2_ in SSFP maximize the SNR, they compromise the spectral resolution.^10^ Additionally, the method’s periodicity introduces a strong offset dependence, resulting in “dark bands” that emit no signal. Hence, only applications involving single lines whose spectral resolution is inconsequential, have reaped the SNR_*t*_ benefits of SSFP. This is widely exploited in MRI;^11−13^ beneficial applications in NQR^14^ and solid-state NMR,^15^ have also been reported. Furthermore, steady-state based applications are emerging in high-resolution solution NMR.^16,17^ This Communication demonstrates that heteronuclear polarization transfers can also be integrated into SSFP, to attain additional SNR_t_ gains for unreceptive, low-γ nuclei.

**Scheme 1:**
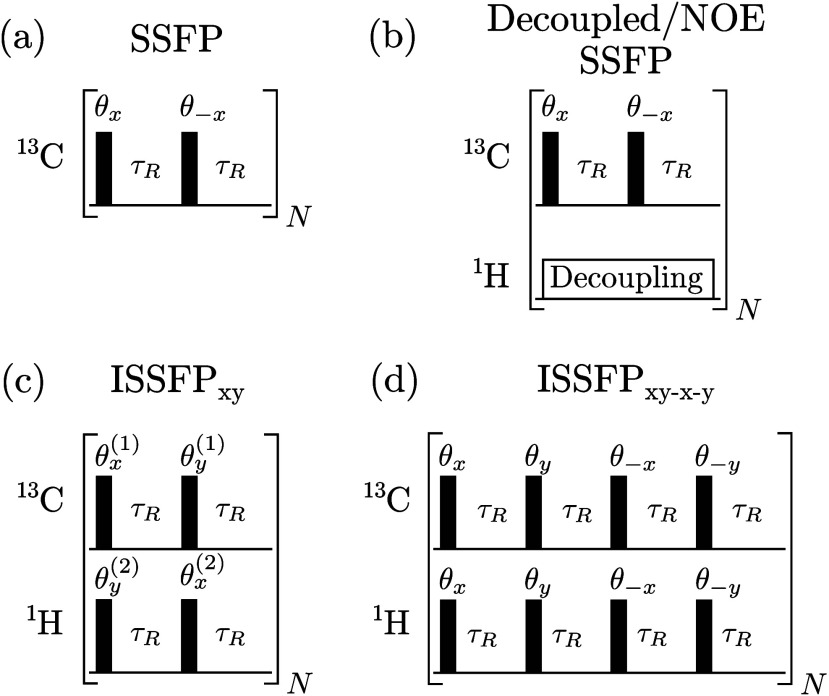
(a) ^13^C SSFP, (b) ^1^H Decoupled/NOE SSFP, (c) ISSFP_*xy*_, and (d) ISSFP_*xy*–*x*–*y*_ Pulse Sequences The filled rectangles represent pulses with flip angle θ and phase as given. τ_R_ is the interpulse delay, which includes the FID acquisition. All sequences assume an on-resonance irradiation on both species.

The scenario discussed here includes a heteronuclear *S* = ^13^C, *I* = ^1^H spin-1/2 pair interacting via *J*-coupling under steady-state conditions—a situation that, to the best of our knowledge, has only been theoretically treated for homonuclear systems.^18^ (a ^1^H and a ^13^C are here assumed, for the sake of simplicity). In analogy to the INEPT experiment, we denote the resulting sequences as INEPT-SSFP (ISSFP), and defer the bulk of their analyses to the Supporting Information. Two sequences which emerged from such analyses are presented in Scheme 1: ISSFP_*xy*_ (Scheme 1c), possessing two blocks of simultaneous ^1^H/^13^C pulses with quadratically incremented phases given by , where *n* is an integer representing each pulse; and ISSFP_*xy*–*x*–*y*_, with four pairs of simultaneous pulses with linear phase increments of π/2 (Scheme 1d). The ^13^C NMR performance of these sequences was evaluated in experiments that compared them to standard SSFP sequences, both with and without continuous ^1^H irradiation; notice that in the latter case, the irradiation produces both decoupling and a ^13^C enhancement due to the nuclear Overhauser effect (NOE).^19^ In addition, optimized INEPT-based ^13^C experiments were also included in the comparison. Representative results of these experiments for a variety of model systems are given in Figure 1 and in Figures S3–S5 of the Supporting Information. Also presented for each case are the corresponding SNR_t_ values, calculated as described in eqs S104–S106 in the Supporting Information from the signals’ maximal intensities and from separate noise-gathering measurements (see insets for each example). Note that the free-induction decay (FID) from each evolution block of the SSFP and ISSFP_*xy*–*x*–*y*_ sequences is the same apart from a well-defined phase shift, and thus the resulting spectra from each block could be coadded after appropriate phase corrections (which can also be done on-the-fly by suitably modulating the receiver phase). By contrast, as further explained below, each of the two evolution blocks of the ISSFP_*xy*_ experiment contributes a different transition of the ^1^H–^13^C doublet to the ^13^C FID; the two blocks thus give rise to peak offsets by the *J*-coupling, and each such spectrum is shown individually in Figures 1 and S3–S5. To ensure a valid assessment, pulse sequence parameters were chosen to maximize the ^13^C sensitivity for each experiment. For the ISSFP_*xy*_ sequence, the optimal repetition time τ_R_ is related to the *J*-coupling constant as τ_R_ = *k*/2*J*, where *k* is an odd integer (see below); for ISSFP_*xy*–*x*–*y*_, the optimal τ_R_ was shorter (τ_R_ < 1/2*J*), and the exact value was determined experimentally. SSFP sequences with ^1^H decoupling exhibited similar SNR_t_ values across a range of τ_R_ (Figures S3–S5). For a given τ_R_, the most suitable flip angle on both channels was then determined experimentally; these match well with predictions from numerical simulations, particularly when RF inhomogeniety was minimized (Figure S6).

**Figure 1:**
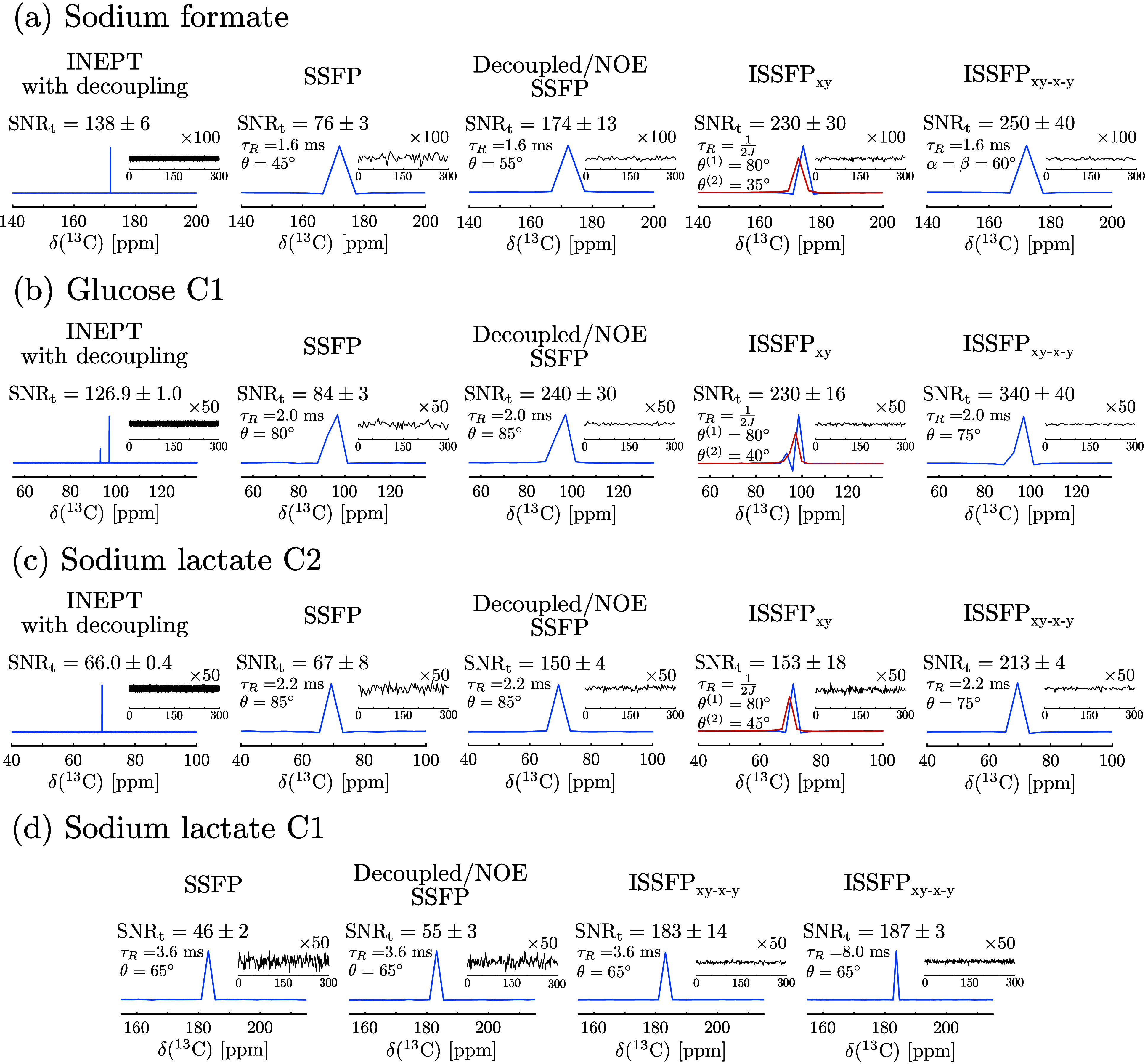
^13^C NMR spectra acquired using different pulse sequences for: (a) sodium formate, (b) glucose C1, (c) sodium lactate C2, and (d) sodium lactate C1. For each spectrum an inset with separate magnified noise is given, which was acquired under the same conditions as the spectrum, but without pulsing. Red and blue spectra for the ISSFP_*xy*_ cases represent outcomes of Fourier processing the first and second (even and odd) blocks, respectively, while the noise shown in the inset is that of the first block. For each experiment, the SNR_*t*_ is shown; for the ISSFP_*xy*_ case, this represents the combined SNR_*t*_ of both blocks. For the steady-state experiments, the chosen repetition time (τ_R_) and pulse flip angle (θ) also are provided; all experiments were executed while on-resonance with the respective ^1^H and ^13^C resonances (in the glucose case, on the majority α-form). For additional experimental details, see the main text and the Supporting Information.

Figure 1a inspects the sensitivity of all sequences for ^13^C spectra of sodium formate in D_2_O—a model with a well-isolated ^1^H/^13^C pair. Both ISSFP sequences show an almost 2-fold boost in SNR_t_ over conventional INEPT, and ca. 3-fold sensitivity increase over conventional SSFP. Spectral resolution in the SSFP experiments is insufficient to resolve the ^13^C doublet, producing a signal intensity akin to that of a decoupled ^13^C resonance. Hence, what is observed as SNR_t_ enhancement upon introducing ^1^H decoupling into SSFP predominantly arises from NOE.^20^ Still, the coherent polarization transfer offered by the ISSFP variants exceed this decoupled SSFP signal by ca. 50%. Figure 1 also presents experiments for solutions of glucose and lactate, metabolites which play a significant role in monitoring tumor metabolism as end reporters of the Warburg effect.^21,22^ The ^1^H/^13^C spin pairs of interest are no longer isolated in these molecules, and more-complex spin dynamics are expected. Furthermore, glucose’s ^13^C resonances are split into α- and β-form peaks, which are not fully resolved by steady-state based experiments. Despite these complications, the ^13^C results arising from the α-form of glucose’s C1 and lactate’s C2 resonances, exhibit similar SNR_t_ trends as seen with formate (Figures 1b and 1c). However, the ISSFP_*xy*–*x*–*y*_ sequence outperforms the ISSFP_*xy*_ experiment, showing a 3-fold to 4-fold enhancement over conventional SSFP and ca. 1.5 times higher SNR_t_ than NOE/decoupled SSFP. By contrast, ISSFP_*xy*_ lacks sensitivity enhancement vis-á-vis SSFP experiments incorporating NOE/decoupling. Interestingly, an efficient polarization transfer for short repetition times also arises from lactate’s carboxylic resonance (Figure 1d). Only a 4 Hz *J*-coupling connects this carbon to ^1^Hs in the molecule (Figure S7), and the increased internuclear distance prevents an efficient NOE enhancement. However, experiments and simulations evidence that the size of the *J*-couplings plays only a secondary role when transferring polarization under ISSFP_*xy–x*–*y*_, steady-state conditions. This bodes well for the enhancement of nonprotonated ^13^C, ^15^N, and ^31^P resonances, particularly with in vivo studies.

In an effort to gain a better understanding of how the ISSFP sequences operate, these were evaluated both analytically and through numerical spin dynamic simulations. The former was carried out by extending the classical single-spin treatment used to derive SSFP conditions,^23,24^ to a two spin-1/2 system interacting by a *J*-coupling. We first examine the ISSFP_*xy*_ sequence, which, although experimentally less efficient than ISSFP_*xy*–*x*–*y*_, has a simpler analysis, revealing insights about the combination of polarization transfer and steady-state processes (see the Supporting Information for details). For simplicity, we focus on θ^(1)^ = θ^(2)^ ≡ θ and τ_*R*_ = 1/2*J* conditions, which, for ISSFP_*xy*_, provide maximal heteronuclear polarization transfer. After each odd set of pulses, the steady-state density operator as a function of flip angle θ is given by
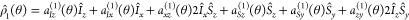
1
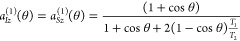
2
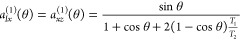
3
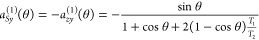
4

In the Cartesian product operator basis,^25^ the density operator consists of six terms (eq 1). These will have equal amplitudes when the pulse flip angle on the two channels is θ = π/2 and *T*_1_ = *T*_2_ (see eqs 2–4), leading to a condition close to maximal transverse magnetization. This is in interesting parallelism to what happens in SSFP for an isolated spin-1/2, where maximal signal intensity is also obtained for such θ and *T*_1_, *T*_2_ conditions, and leads to a steady-state magnetization that is evenly distributed over longitudinal and transverse terms.^24^ Note that the relation governing the transverse term dependence on the flip angle θ (see eqs 3 and 4) is also analogous to that in conventional SSFP.^24^ Additionally, in parallel to SSFP experiments, the ISSFP signal intensity within these approximations depends on the *T*_1_/*T*_2_ ratio but not on absolute *T*_1_ and *T*_2_ values. ISSFP, however, has its distinctive features: both coupled spins will now share their polarizations equally (as expected due to the sequence’s symmetry), and each of these will be associated with three terms: a longitudinal and an in-phase transverse magnetization as in the isolated spin-1/2 case, plus additional antiphase transverse terms originating from the *J*-coupling. All of these will share in 1/6 of the total initial spin order, which, for simplicity, we assumed to be equal for *I* and *S*. The spectra that arise from the combination of the in-phase and antiphase transverse terms in eqs 3 and 4 may be better appreciated in the fictitious spin-1/2 basis^26^ (see eqs S80 and S81). For instance, the sum of  and  leads to only one of the two observable transitions for the *S*-spin; the same occurs with the transverse *I*-spin terms. Hence, for both spins, their resulting spectra will consist of a single peak out of each *J*-doublet. A similar analysis for the steady-state density operator after the even set of pulses , gives combined operators that originate from the other transition for each spin, i.e., the second multiplet component of each spin’s *J*-coupled signal. A schematic summary of these findings is presented in Figures 2a and 2b. This is in agreement with the experimentally observed resonance shifts arising from the first and second evolution periods of the ISSFP_*xy*_ sequence, as exemplified in Figures 1 and S3–S5.

**Figure 2:**
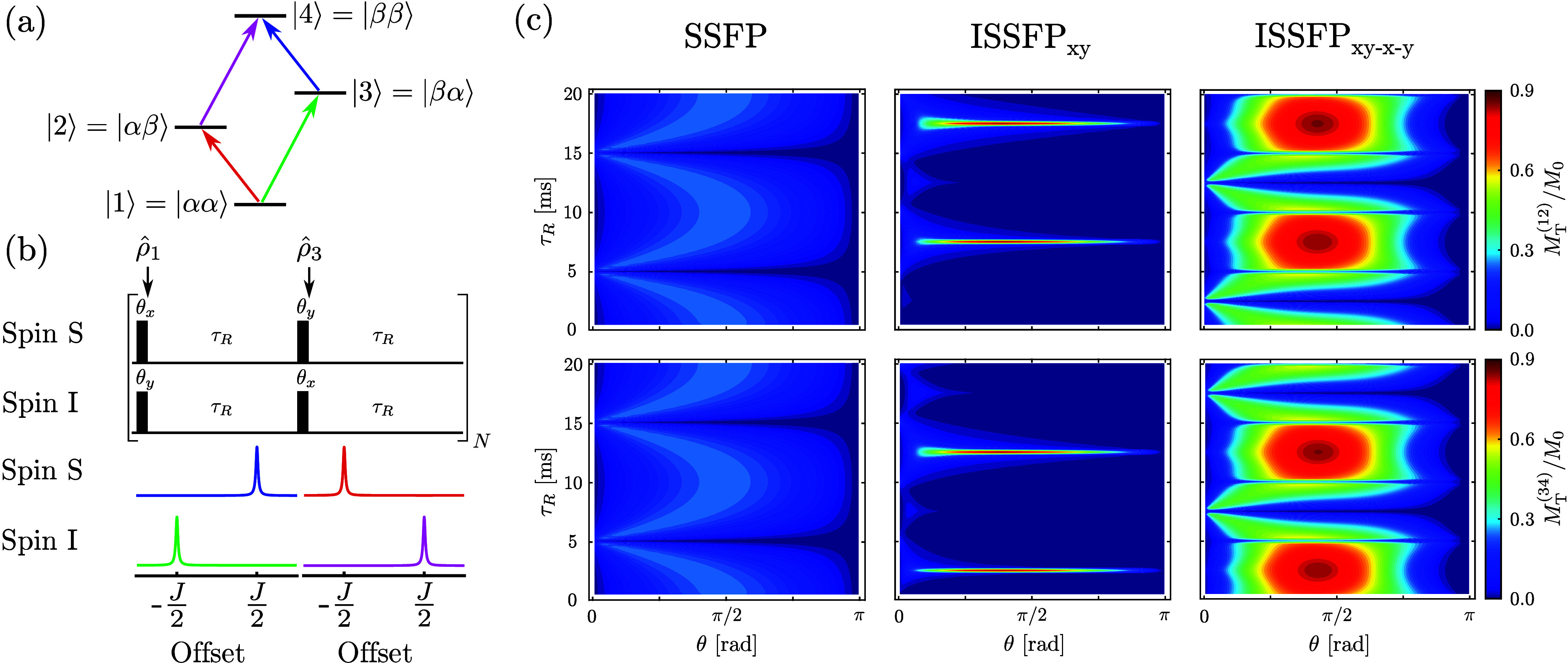
(a) Energy level diagram of a two spin–1/2 system *I* – *S*, assuming that ω_*I*_ > ω_*S*_ ≫ *J* > 0. Here |*m*_*I*_*m*_*S*_⟩ represent the eigenstates of Zeeman and heteronuclear *J*-coupling Hamiltonian in the high-field approximation, with *m*_*N*_ being the magnetic quantum number and ω_*N*_ being the Larmor frequency for each spin. The allowed values of *m*_*N*_ are labeled as α and β. The arrows connecting the energy levels (1 through 4) represent the observable transitions for each spin: red (1 → 2) and blue (3 → 4) for spin *S*, and green (1 → 3) and purple (2 → 4) for spin *I*. (b) Schematic ISSFP_*xy*_ sequence with the resulting spectra arising from each block for both spins, and with spectral colors associated with the transitions in panel (a). Spectra are referenced to the Larmor frequency of each spin. Density operators  and  describe the state of the spin ensemble at the time points marked by black arrows. (c) Simulations of the steady-state ^13^C transverse magnetization at the beginning of the first block (within ) of the indicated sequences as a function of repetition times τ_R_ and flip angle θ. Notice that separate plots are provided for each of the two observable *S* transitions (1 → 2 and 3 → 4). Spins *I* and *S* are chosen as ^1^H and ^13^C, respectively, with a *J*-coupling of 200 Hz. Additional simulation details are given in the Supporting Information.

Extending such examination to the θ = π/2 scenario yields the signal dependence of ISSFP_*xy*_ on repetition time τ_R_. Transverse magnetization maxima will then occur whenever τ_R_ = *k*/2*J*, where *k* is an odd integer (eqs S84–S95). Furthermore, the set of operators that evolve during the first and second periods alternate between the maxima. For instance, from the odd blocks, spectra will consist of peaks arising from transitions 1 → 3 and 3 → 4 for *k* = 1, 5, 9, ..., while operators 2 → 4 and 1 → 2 will originate the maximal signals for *k* = 3, 7, 11, ... These operators will swap conditions for signals arising from the even ISSFP_*xy*_ blocks. These predictions agree with the signal intensities predicted by numerical simulations (Figure 2c) and with the experimental data, which show that observed resonance switches between multiplet components for the odd and even blocks of ISSFP_*xy*_ experiments conducted with τ_R_ of 1/2*J* and 3/2*J* (Figures S3–S5).

An analytical examination of the ISSFP_*xy*–*x*–*y*_ sequence is more complex; nevertheless, a relatively simple steady-state density operator solution is found as a function of τ_R_ (eqs S96–S103). The solution contains more transverse operators than in the ISSFP_*xy*_ case. Some of the operator amplitudes do not depend on the *J*-coupling; hence, these terms are void of an oscillatory dependence on τ_R_. Still, the observable transition operators behave in a similar, alternate manner as they do in the simpler ISSFP_*xy*_ variant. Figure 3 illustrates this using, once again, sodium formate as a prototypical model, evidencing—in addition to a clear signal enhancement—oscillatory τ_R_-dependent features in the ISSFP_*xy*–*x*–*y*_ variant. Some of these are also evident in data collected using ^1^H-coupled ^13^C SSFP experiments, which reflect the off-resonance effects introduced by the *J*-coupling. Notice that the positions of the “dark bands” in all these curves will be solely dependent on τ_R_, even if their overall shapes will naturally be dependent on the flip angles applied on both species. Notice as well that since transverse terms exist even when using short repetition times τ_R_ < 1/2*J*, efficient polarization transfers occur even for small *J*-couplings. This is evident in the ISSFP_*xy*–*x*–*y*_ curves in Figure 3, and also explains the surprisingly good enhancement observed for lactate’s ^13^C carboxyl site (Figure 1d). Notice that numerical simulations (Figure 2c) substantiate the analytical results outlined above. For instance, they reveal that maxima for the individual transitions occur at τ_R_ = *k*/2*J* with *k* = 1, 5, ... for transition 3 → 4, while with *k* = 3, 7, ... for transition 1 → 2.

**Figure 3:**
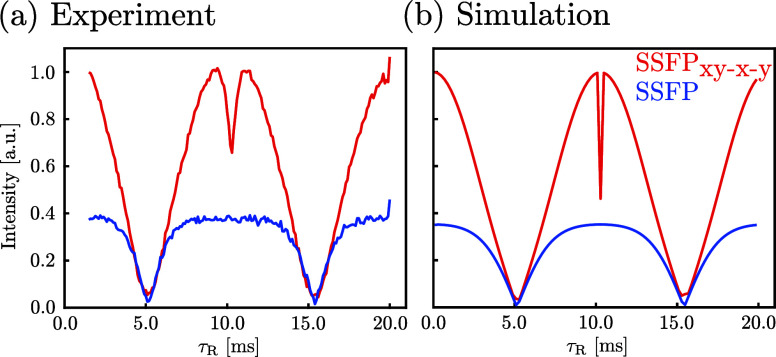
(a) Experimental and (b) simulated steady-state ^13^C transverse magnetization observed for sodium formate, as a function of the repetition time τ_R_, from the SSFP (blue) and ISSFP_*xy*–*x*–*y*_ (red) sequences. The dashed lines mark the “dark band” positions of the ISSFP_*xy*–*x*–*y*_ sequence. The plotted signals correspond in all cases to the point of the FID located at τ_R_/2. This highlights the “dark band” positions; however, it does not reflect all the maxima, due to destructive interference between the signals from the two transitions. Sample and conditions were identical in both experiments and as detailed in Figure 1a. Simulations were carried out using the experimental parameters, for a spin system comprised of two spins (^1^H and ^13^C) with *J* = 195 Hz. All transverse relaxation times were assumed 4 s, *T*_1_^H^ = 13.5 s and *T*_1_^C^ = 20 s. See the Supporting Information for additional details.

The theoretical analysis above also gives qualitative insight into the maximal enhancements that the new ISSFP sequences will achieve over conventional SSFP counterparts. Taking into account that (i) the full polarization of the *I* and *S* spins (proportional to γ_*S*_ + γ_*I*_) will, in ISSFP’s best-case scenario, be equally split among six spin operators–two transverse and one longitudinal for each spin; (ii) that in conventional SSFP will, in an equal best case scenario, yield a maximal signal proportional to γ_*S*_/2; then, a maximum enhancement value η_*S*_ can be computed for the *S*-spin signal as
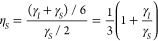
5

According to this relation, *S* = ^13^C ISSFP sequences involving polarization transferred from *I* = ^1^H should provide a maximal enhancement of ca. 1.7 times over SSFP when all components in the density operator relax with the same rate. Experimental observations, however, (Figures 1, 3, and S3–S5) reveal up to 4-fold improvement in SNR_t_ compared to conventional SSFP. Simulations show that this additional enhancement is associated with the unequal relaxation times of the two species, in particular to the fact that the *T*_1_/*T*_2_ ratio for ^13^C is larger than that for ^1^H. This boosts the efficiency of the ISSFP sequences over SSFP counterparts (Figure S8a). The experimental enhancements of ISSFP_*xy*_ match very well with predictions of this model (Figure S8b), particularly when taking into account that increasing the ^1^H *T*_1_/*T*_2_ ratio seems to have an opposite effect on the relative enhancement. Still, a complete understanding of the experimental enhancement requires the measurement and incorporation of a complete set of relaxation parameters, including cross-relaxation and cross-correlation effects that have no parallel in the isolated-spin SSFP scenario. As suggested by eq 5, ISSFP’s advantages will improve with decreasing γ_S_ and, as mentioned, these gains will also be present in cases of long-range ^1^H couplings, where NOE enhancements become negligible.

One of the applications envisioned for these new sequences is in metabolic low-γ nuclide MRI, where SSFP sequences have proven well-suited to enhance the sensitivity of the sparse spectra involved.^27,28^ To explore this aspect, experiments were tested using a phantom of d-glucose-^13^C1 in 2% agarose. Figure 4 demonstrates the ca. 3-fold gain in intensity that ISSFP_*xy*–*x*–*y*_ can provide over SSFP variant in the ensuing images, and the 50% signal enhancement that yields over ^13^C SSFP MRI with continuous ^1^H irradiation. These trends, as well as the flip-angle dependence, are all in excellent agreement with the spectroscopic data and the theoretical assessment.

**Figure 4:**
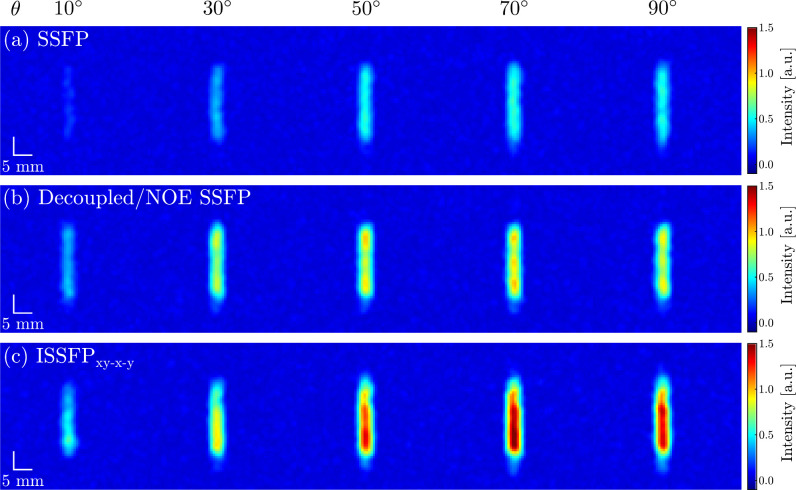
^13^C images of 50 mM d-glucose-^13^C1 dissolved in a 5-mm-diameter 2% agarose/H_2_O phantom acquired with (a) SSFP, (b) SSFP with ^1^H decoupling and NOE and (c) ISSFP_*xy*–*x*–*y*_ sequences. From left to right, the images are shown as a function of the pulse flip angle θ in the sequences. See the Supporting Information for additional details.

The present study demonstrated the feasibility of coupling INEPT with SSFP, to achieve a coherent polarization transfer under steady-state conditions. To this end, a sequence of lower experimental performance but simpler interpretation was introduced (ISSFP_*xy*_), as was a more efficient but theoretically more-complex sequence (ISSFP_*xy*–*x*–*y*_). Notice that this extra complexity is not translated experimentally, as the sequence’s phase incrementation simply represents pulsing on both channels with constant phases at an offset ΔΩ = π/2τ_R_. Theoretical analyses, simulations, and experiments demonstrated that substantial improvements in SNR_t_ could then be obtained in comparison to either conventional INEPT or SSFP acquisitions. When executed within an NMR framework the experiment is thus simple; notice that, since the duty cycles of the ensuing sequences are 0.1%, even a reliance on pulses with hundreds of watts of power will lead to an average heat deposition lower than that of typical continuous decoupling sequences; all these experiments are thus compatible with cryogenically cooled probes. These spectroscopic gains translated also into thermal ^13^C MRI experiments, and could provide a valuable complement to hyperpolarized MRSI.^29,30^ Similarly to what happens with other SSFP experiments, the new ISSFP sequences exhibit a sensitivity to resonance offset that can lead to appearance of “dark bands” (Figure S9); we have observed these in ^13^C 15.2T ISSFP_*xy*–*x*–*y*_ images, with blind spots tracking the patterns observed for ^1^H SSFP NMR images collected with the same τ_R_. Ongoing studies show that these inhomogeneities are of secondary importance at the low field strengths of relevance in potential clinical MRSI applications. The offset dependence will also complicate ISSFP’s uses as a broadband approach to enhance arbitrary offsets with a fixed phase-incrementation scheme; nevertheless, pulse sequence parameters that enhance the signal of multiple resonances can be found (see Figure S10). However, a more robust approach for achieving enhancement with arbitrary offsets could be obtained if combined with the recently introduced phase incremented SSFP schemes.^17^ Alternatively, broadband performance can be achieved by relying on a priori information about the peaks being targeted, as is done in SSFP-based spectroscopic imaging experiments.^31,32^ The sequences described herein also open the way for evaluating variants tackling systems with multiple heteronuclear *J*-couplings, and eventually homonuclear *J*-couplings. They could also carry the seeds for developing new forms multidimensional correlative NMR based on steady-state experiments. These investigations are in progress.

## Supporting Information Available

Materials and methods used; MRI pulse sequences; operator analysis of the ISSFP sequences; additional experimental results (effects of offset, RF inhomogeneity, signal dependence on flip angle); additional simulations; theoretical maxima for various species (PDF)
