# Supplementary material for: Heteronuclear Polarization Transfer under Steady-State Conditions: The INEPT-SSFP Experiment
Source: J Phys Chem Lett. 2024 Oct 16;15(42):10644–50. doi: 10.1021/acs.jpclett.4c02016 (PMC11514021; doi:10.1021/acs.jpclett.4c02016)
Supplement: Supplementary file 1 — jz4c02016_si_001.pdf [file jz4c02016_si_001.pdf]

Supporting information for "Heteronuclear polarization transfer under steady-state conditions: The INEPT-SSFP experiment"

Rihards Aleksis, Elton T. Montrazi, and Lucio Frydman*

Department of Chemical and Biological Physics, Weizmann Institute, 7610001 Rehovot, Israel

E-mail: lucio.frydman@weizmann.ac.il

Theoretical background

Basis operators

Here we review various operator bases that are convenient for representing the spin dynamics of the sequences described in this study. We consider a two spin-1/2 system $I - S$. The density operator of such system can be expressed as a linear combination of 16 base operators. If defined using Ernst's Cartesian product operator basis[1] these include, apart from the identity operator $\frac{1}{2}\hat{E}$, longitudinal magnetization operators $\hat{I}_z$, $\hat{S}_z$, in-phase x,y-magnetization operators $\hat{I}_x, \hat{I}_y, \hat{S}_x, \hat{S}_y$, and anti-phase x, y-magnetization operators $2\hat{I}_x\hat{S}_z$, $2\hat{I}_y\hat{S}_z$, $2\hat{I}_z\hat{S}_x$, $2\hat{I}_z\hat{S}_y$. Additionally, there are product operators corresponding to two spin coherences: $2\hat{I}_x\hat{S}_x$, $2\hat{I}_x\hat{S}_y$, $2\hat{I}_y\hat{S}_x$, $2\hat{I}_y\hat{S}_y$, and a longitudinal two spin order $2\hat{I}_z\hat{S}_z$.

A second basis we use in this discussion is the fictitious spin-1/2 operator basis proposed by Vega.[2] In general, for a spin system with n eigenstates $|k\rangle$, where $k = 1, 2, \ldots n$, the

fictitious spin-1/2 operators for a pair of states $|i\rangle$ and $|j\rangle$ are given by:

$$\hat{I}_z^{(ij)} = \frac{1}{2}\left(|i\rangle\langle i| - |j\rangle\langle j|\right), \tag{S1}$$

$$\hat{I}_x^{(ij)} = \frac{1}{2}\left(|i\rangle\langle j| + |j\rangle\langle i|\right), \tag{S2}$$

$$\hat{I}_y^{(ij)} = \frac{1}{2i}\left(|i\rangle\langle j| - |j\rangle\langle i|\right), \tag{S3}$$

$$\hat{E}^{(ij)} = |i\rangle\langle i| + |j\rangle\langle j| \tag{S4}$$

In analogy to Pauli's matrices, these spin angular momentum operators follow the cyclic commutation relationships $\left[\hat{I}_x^{(ij)}, I_y^{(ij)}\right] = i\hat{I}_z^{(ij)}$. For a two spin system as defined in the main text (Figure 2b) we have 4 such eigenstates $|n\rangle$, i.e., $|1\rangle = |\alpha\alpha\rangle$, $|2\rangle = |\alpha\beta\rangle$, $|3\rangle| = |\beta\alpha\rangle$ and $|4\rangle = |\beta\beta\rangle$, where $\alpha = 1/2$ and $\beta = -1/2$ represent the magnetic quantum numbers. We summarize a few relations between the two bases that later we will find useful:

$$\hat{I}_z^{(14)} = \hat{I}_z + \hat{S}_z, \tag{S5}$$

$$\hat{I}_y^{(34)} = \frac{1}{2}\left(\hat{S}_y - 2\hat{I}_z\hat{S}_y\right), \tag{S6}$$

$$\hat{I}_x^{(12)} = \frac{1}{2}\left(\hat{S}_x - 2\hat{I}_z\hat{S}_x\right), \tag{S7}$$

$$\hat{I}_x^{(13)} = \frac{1}{2}\left(\hat{I}_x - 2\hat{I}_x\hat{S}_z\right), \tag{S8}$$

$$\hat{I}_y^{(24)} = \frac{1}{2}\left(\hat{I}_y - 2\hat{I}_y\hat{S}_z\right). \tag{S9}$$

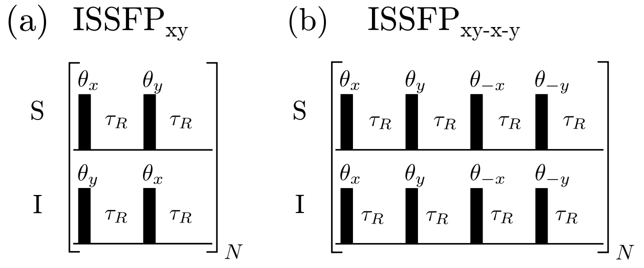

Figure S1: ISSFP_{xy} (a) and $\text{ISSFP}_{\text{xy-x-y}}$ (b) pulse sequences. The filled rectangles represent radiofrequency pulses with flip angle θ and phases are given in Cartesian coordinates. τ_{R} is the interpulse delay, which includes the FID acquisition time.

Analysis of the ISSFP$_{\mathbf{xy}}$ sequence

In order to simplify the analysis we examine this case when the repetition time is set to $\tau_{\mathrm{R}} = 1/2J$. We assume that a steady-state has been achieved by repeating N SSFP cycles; then, at the beginning of the $(N+1)^{\text{th}}$ cycle (end of the Nth cycle), which is immediately before the cycle's first (odd) set of pulses the density operator as a function of flip angle θ is given by:

$$\begin{aligned}\hat{\rho}_0(\theta) =& a_{Iz}^{(0)}(\theta)\hat{I}_z + a_{Ix}^{(0)}(\theta)\hat{I}_x + a_{Iy}^{(0)}(\theta)\hat{I}_{Iy} + a_{Sz}^{(0)}(\theta)\hat{S}_z + a_{Sx}^{(0)}(\theta)\hat{S}_x \\ &+ a_{Sy}^{(0)}(\theta)\hat{S}_y + a_{xz}^{(0)}(\theta)2\hat{I}_x\hat{S}_z + a_{yz}^{(0)}(\theta)2\hat{I}_y\hat{S}_z + a_{zx}^{(0)}(\theta)2\hat{I}_z\hat{S}_x + a_{zy}^{(0)}(\theta)2\hat{I}_z\hat{S}_y \\ &+ a_{zz}^{(0)}(\theta)2\hat{I}_z\hat{S}_z + a_{xy}^{(0)}(\theta)2\hat{I}_x\hat{S}_y + a_{yx}^{(0)}(\theta)2\hat{I}_y\hat{S}_x + a_{xx}^{(0)}(\theta)2\hat{I}_x\hat{S}_x + a_{yy}^{(0)}(\theta)2\hat{I}_y\hat{S}_y. \end{aligned} \tag{S10}$$

The identity operator was ignored here, as it commutes with all other terms. For simplicity we will write the operator amplitudes as $a_{kl}^{(n)}(\theta) \equiv a_{kl}^{(n)}$. After the $(N+1)^{\text{th}}$ cycle's first (odd) set of pulses we define the density operator as $\hat{\rho}_1(\theta)$ (Fig.2b), and Cartesian product operators are proportional to the following set of coefficients $\left\{a_{1z}^{(1)}, a_{Ix}^{(1)}, a_{Iy}^{(1)}, a_{Sz}^{(1)}, a_{Sx}^{(1)}, a_{Sy}^{(1)}, a_{xz}^{(1)}, a_{yz}^{(1)}, a_{zx}^{(1)}, a_{zy}^{(1)}, a_{zz}^{(1)}, a_{xy}^{(1)}, a_{yx}^{(1)}, a_{xx}^{(1)}, a_{yy}^{(1)}\right\}$, which are given by:

$$a_{Iz}^{(1)} = a_{Iz}^{(0)}\cos\theta - a_{Ix}^{(0)}\sin\theta, \tag{S11}$$

$$a_{Ix}^{(1)} = a_{Ix}^{(0)}\cos\theta + a_{Iz}^{(0)}\sin\theta, \tag{S12}$$

$$a_{Iy}^{(1)} = a_{Iy}^{(0)}, \tag{S13}$$

$$a_{Sz}^{(1)} = a_{Sz}^{(0)}\cos\theta + a_{Sy}^{(0)}\sin\theta, \tag{S14}$$

$$a_{Sx}^{(1)} = a_{Sx}^{(0)}, \tag{S15}$$

$$a_{Sy}^{(1)} = a_{Sy}^{(0)}\cos\theta - a_{Sz}^{(0)}\sin\theta, \tag{S16}$$

$$a_{xz}^{(1)} = a_{xz}^{(0)}\cos^2\theta + \frac{1}{2},\left(a_{xy}^{(0)} + a_{zz}^{(0)}\right)\sin 2\theta + a_{zy}^{(0)}\sin^2\theta, \tag{S17}$$

$$a_{yz}^{(1)} = a_{yz}^{(0)}\cos\theta + a_{yy}^{(0)}\sin\theta, \tag{S18}$$

$$a_{zx}^{(1)} = a_{zx}^{(0)}\cos^2\theta - a_{xx}^{(0)}\sin\theta, \tag{S19}$$

$$a_{zy}^{(1)} = a_{zy}^{(0)}\cos^2\theta - \frac{1}{2},\left(a_{xy}^{(0)} + a_{zz}^{(0)}\right)\sin 2\theta + a_{xz}^{(0)}\sin^2\theta, \tag{S20}$$

$$a_{zz}^{(1)} = a_{zz}^{(0)} \cos^2\theta - \frac{1}{2}\left(a_{xz}^{(0)} - a_{zy}^{(0)}\right)\sin 2\theta - a_{xy}^{(0)} \sin^2\theta, \tag{S21}$$

$$a_{xy}^{(1)} = a_{xy}^{(0)} \cos^2\theta - \frac{1}{2}\left(a_{xz}^{(0)} - a_{zy}^{(0)}\right)\sin 2\theta - a_{zz}^{(0)} \sin^2\theta, \tag{S22}$$

$$a_{yx}^{(1)} = a_{yx}^{(0)}, \tag{S23}$$

$$a_{xx}^{(1)} = a_{xx}^{(0)} \cos\theta + a_{zx}^{(0)} \sin\theta, \tag{S24}$$

$$a_{yy}^{(1)} = a_{yy}^{(0)} \cos\theta - a_{yz}^{(0)} \sin\theta. \tag{S25}$$

After $(N+1)^{\text{th}}$ cycle's first (odd) free evolution period ($\tau_{\text{R}} = \frac{1}{2J}$) these coefficients are:

$$a_{Iz}^{(2)} = 1 - E_1 + a_{Iz}^{(1)} E_1, \tag{S26}$$

$$a_{Ix}^{(2)} = -a_{yz}^{(1)} E_2, \tag{S27}$$

$$a_{Iy}^{(2)} = a_{xz}^{(1)} E_2, \tag{S28}$$

$$a_{Sz}^{(2)} = 1 - E_1 + a_{Sz}^{(1)} E_1, \tag{S29}$$

$$a_{Sx}^{(2)} = -a_{zy}^{(1)} E_2, \tag{S30}$$

$$a_{Sy}^{(2)} = a_{zx}^{(1)} E_2, \tag{S31}$$

$$a_{xz}^{(2)} = -a_{Iy}^{(1)} E_2, \tag{S32}$$

$$a_{yz}^{(2)} = a_{Ix}^{(1)} E_2, \tag{S33}$$

$$a_{zx}^{(2)} = -a_{Sy}^{(1)} E_2, \tag{S34}$$

$$a_{zy}^{(2)} = a_{Sx}^{(1)} E_2, \tag{S35}$$

$$a_{zz}^{(2)} = a_{zz}^{(1)} E_1, \tag{S36}$$

$$a_{xy}^{(2)} = a_{xy}^{(1)} E_2, \tag{S37}$$

$$a_{yx}^{(2)} = a_{yx}^{(1)} E_2, \tag{S38}$$

$$a_{xx}^{(2)} = a_{xx}^{(1)} E_2, \tag{S39}$$

$$a_{yy}^{(2)} = a_{yy}^{(1)} E_2, \tag{S40}$$

with relaxation included here phenomenologically by $E_1 = \exp(-\tau_{\text{R}}/T_1)$ and $E_2 = \exp(-\tau_{\text{R}}/T_2)$. Every longitudinal component is assumed to relax with constant T_1, while any transverse component, including anti-phase and multi spin coherences, will relax with constant T_2. We

also assume that both spins have equal equilibrium polarization. After the second (even) set of pulses the density operator $\hat{\rho}_3(\theta)$ (Fig. 2b) is defined as a linear combination of Cartesian product operators with amplitudes:

$$a_{Iz}^{(3)} = \cos\theta + \left(a_{Iz}^{(1)} - 1\right) E_1 \cos\theta + a_{xz}^{(1)} E_2 \sin\theta, \tag{S41}$$

$$a_{Ix}^{(3)} = -a_{yz}^{(1)} E_2, \tag{S42}$$

$$a_{Iy}^{(3)} = a_{xz}^{(1)} E_2 \cos\theta + \left(E_1 - 1 - E_1 a_{Iz}^{(1)}\right) \sin\theta, \tag{S43}$$

$$a_{Sz}^{(3)} = \cos\theta + \left(a_{Sz}^{(1)} - 1\right) E_1 \cos\theta + a_{zy}^{(1)} E_2, \tag{S44}$$

$$a_{Sx}^{(3)} = -a_{zy}^{(1)} E_2 \cos\theta + \sin\theta + E_1 (a_{Sz}^{(1)} - 1) \sin\theta, \tag{S45}$$

$$a_{Sy}^{(3)} = a_{zx}^{(1)} E_2, \tag{S46}$$

$$a_{xz}^{(3)} = -\left(\cos\theta a_{Iy}^{(1)} + \sin\theta a_{xx}^{(1)}\right) E_2, \tag{S47}$$

$$a_{yz}^{(3)} = a_{Ix}^{(1)} E_2 \cos^2\theta - \frac{1}{2}\left(E_2 a_{yx}^{(1)} + E_1 a_{zz}^{(1)}\right) \sin 2\theta - a_{Sy}^{(1)} E_2 \sin^2\theta, \tag{S48}$$

$$a_{zx}^{(3)} = -a_{Sy}^{(1)} E_2 \cos^2\theta + \frac{1}{2}\left(E_2 a_{yx}^{(1)} + E_1 a_{zz}^{(1)}\right) \sin 2\theta + a_{Ix}^{(1)} E_2 \sin^2\theta, \tag{S49}$$

$$a_{zy}^{(3)} = \left(\cos\theta a_{Sx}^{(1)} + \sin\theta a_{yy}^{(1)}\right) E_2, \tag{S50}$$

$$a_{zz}^{(3)} = a_{zz}^{(1)} E_1 \cos^2\theta + \frac{1}{2}\left(E_2 a_{Ix}^{(1)} + E_1 a_{Sy}^{(1)}\right) \sin 2\theta - a_{yx}^{(1)} E_2 \sin^2\theta, \tag{S51}$$

$$a_{xy}^{(3)} = a_{xy}^{(1)} E_2, \tag{S52}$$

$$a_{yx}^{(3)} = a_{yx}^{(1)} E_2 \cos^2\theta + \frac{1}{2}\left(a_{1x}^{(1)} + a_{Sy}^{(1)}\right) E_2 \sin 2\theta - a_{zz}^{(1)} E_1 \sin^2\theta, \tag{S53}$$

$$a_{xx}^{(3)} = \left(\cos\theta a_{xx}^{(1)} - \sin\theta a_{Iy}^{(1)}\right) E_2, \tag{S54}$$

$$a_{yy}^{(3)} = \left(\cos\theta a_{yy}^{(1)} - \sin\theta a_{Sx}^{(1)}\right) E_2. \tag{S55}$$

Finally, after the second (even) free evolution period ($\tau_{\mathrm{R}} = \frac{1}{2J}$) the amplitudes are given by:

$$a_{Iz}^{(4)} = 1 - E_1 + E_1 \cos\theta \left(1 + E_1 \left(a_{Iz}^{(1)} - 1\right)\right) + E_1 E_2 \sin\theta a_{xz}^{(1)}, \tag{S56}$$

$$a_{Ix}^{(4)} = \frac{1}{2} E_2 \left(a_{2y} \left(1 - \cos 2\theta\right) E_2 - a_{1x} \left(1 + \cos 2\theta\right) E_2 + \left(a_{yx}^{(1)} E_2 + a_{zz}^{(1)} E_1\right) \sin 2\theta\right), \tag{S57}$$

$$a_{Iy}^{(4)} = -E_2^2 \left(\cos\theta a_{Iy}^{(1)} + \sin\theta a_{xx}^{(1)}\right), \tag{S58}$$

$$a_{Sz}^{(4)} = 1 - E_1 + E_1 \cos\theta \left(1 + E_1 \left(a_{Sz}^{(1)} - 1\right)\right) + E_1 E_2 \sin\theta a_{zy}^{(1)}, \tag{S59}$$

$$a_{Sx}^{(4)} = -E_2^2 \left(a_{Sx}^{(1)} \cos\theta + a_{yy}^{(1)} \sin\theta\right), \tag{S60}$$

$$a_{Sy}^{(4)} = \frac{1}{2} E_2 \left(a_{Ix} \left(1 - \cos 2\theta\right) E_2 - a_{Sy} \left(1 + \cos 2\theta\right) E_2 + \left(a_{xy}^{(1)} E_2 + a_{zz}^{(1)} E_1\right) \sin 2\theta\right), \tag{S61}$$

$$a_{xz}^{(4)} = E_2 \left(\sin\theta - a_{xz}^{(1)} E_2 \cos\theta + \left(a_{Iz}^{(1)} - 1\right) E_1 \sin\theta\right), \tag{S62}$$

$$a_{yz}^{(4)} = -a_{yz}^{(1)} E_2^2, \tag{S63}$$

$$a_{zx}^{(4)} = -a_{zx}^{(1)} E_2^2, \tag{S64}$$

$$a_{zy}^{(4)} = E_2 \left(\sin\theta - a_{zy}^{(1)} E_2 \cos\theta + \left(a_{Sz}^{(1)} - 1\right) E_1 \sin\theta\right), \tag{S65}$$

$$a_{zz}^{(4)} = \frac{1}{2} E_1 \left(a_{zz}^{(1)} \left(1 + \cos 2\theta\right) E_1 - a_{yx}^{(1)} \left(1 - \cos 2\theta\right) E_2 + \left(a_{Ix}^{(1)} + a_{Sy}^{(1)}\right) E_2 \sin 2\theta\right), \tag{S66}$$

$$a_{xy}^{(4)} = E_2^2 a_{xy}^{(1)}, \tag{S67}$$

$$a_{yx}^{(4)} = \frac{1}{2} E_2 \left(a_{yx}^{(1)} \left(1 + \cos 2\theta\right) E_2 - a_{zz}^{(1)} \left(1 - \cos 2\theta\right) E_1 + \left(a_{Ix}^{(1)} + a_{Sy}^{(1)}\right) E_2 \sin 2\theta\right), \tag{S68}$$

$$a_{xx}^{(4)} = E_2^2 \left(\cos\theta a_{xx}^{(1)} - \sin\theta a_{Iy}^{(1)}\right), \tag{S69}$$

$$a_{yy}^{(4)} = E_2^2 \left(\cos\theta a_{yy}^{(1)} - \sin\theta a_{Sx}^{(1)}\right). \tag{S70}$$

If a steady-state is formed then the density operator is periodic over the $\mathrm{ISSFP_{xy}}$ sequence, and thus $\hat{\rho}_0(\theta) \equiv \hat{\rho}_4(\theta)$. Here $\hat{\rho}_4(\theta)$ is density operator at the end second evolution period and is expressed as:

$$\begin{aligned}\hat{\rho}_4(\theta) =& a_{Iz}^{(4)}(\theta)\hat{I}_z + a_{Ix}^{(4)}(\theta)\hat{I}_x + a_{Iy}^{(4)}(\theta)\hat{I}_{Iy} + a_{Sz}^{(4)}(\theta)\hat{S}_z + a_{Sx}^{(4)}(\theta)\hat{S}_x \\ &+ a_{Sy}^{(4)}(\theta)\hat{S}_y + a_{xz}^{(4)}(\theta)2\hat{I}_x\hat{S}_z + a_{yz}^{(4)}(\theta)2\hat{I}_y\hat{S}_z + a_{zx}^{(4)}(\theta)2\hat{I}_z\hat{S}_x + a_{zy}^{(4)}(\theta)2\hat{I}_y\hat{S}_z \\ &+ a_{zz}^{(4)}(\theta)2\hat{I}_z\hat{S}_z + a_{xy}^{(4)}(\theta)2\hat{I}_x\hat{S}_y + a_{yx}^{(4)}(\theta)2\hat{I}_y\hat{S}_x + a_{xx}^{(4)}(\theta)2\hat{I}_x\hat{S}_x + a_{yy}^{(4)}(\theta)2\hat{I}_y\hat{S}_y.\end{aligned} \tag{S71}$$

Since the Cartesian product operator basis is orthogonal, each product operator amplitude must be equal in the two density operators ($\hat{\rho}_0$ and $\hat{\rho}_4$), which leads to a system of linear equations. Using Eqs. S11-S25 the coefficients $a_{kl}^{(0)}$ can be expressed as a combination of amplitudes $a_{kl}^{(1)}$. These relations, together with Eqs. S56-S70, can be substituted into the system of linear equations, which is then given in matrix form as:

$$
\begin{pmatrix}
E_1^2C^2-1 & \frac{1}{2}E_2^2S\,(1+C_2) & 0 & 0 & 0 & \frac{1}{2}E_2^2S\,(C_2-1) & E_1E_2CS & 0 & 0 & 0 & -\frac{1}{2}E_1E_2SS_2 & 0 & \frac{1}{2}E_2^2SS_2 & 0 & 0 \\
E_1^2CS & \frac{1}{2}E_2^2C\,(1+C_2)-1 & 0 & 0 & 0 & \frac{1}{2}E_2^2C\,(1-C_2) & E_1E_2S^2 & 0 & 0 & 0 & \frac{1}{2}E_1E_2CS_2 & 0 & \frac{1}{2}E_2^2CS_2 & 0 & 0 \\
0 & 0 & -1-E_2^2C & 0 & 0 & 0 & 0 & 0 & 0 & 0 & 0 & 0 & 0 & -E_2^2S & 0 \\
0 & \frac{1}{2}E_2^2S\,(1-C_2) & 0 & E_1^2C^2-1 & 0 & \frac{1}{2}E_2^2S\,(1+C_2) & 0 & 0 & 0 & E_1E_2CS & \frac{1}{2}E_1E_2SS_2 & 0 & \frac{1}{2}E_2^2SS_2 & 0 & 0 \\
0 & 0 & 0 & 0 & E_2^2C-1 & 0 & 0 & 0 & 0 & 0 & 0 & 0 & 0 & 0 & -\frac{1}{2}E_2^2S \\
0 & \frac{1}{2}E_2^2C\,(1-C_2) & 0 & E_1^2CS & 0 & \frac{1}{2}E_2^2C\,(1+C_2)-1 & 0 & 0 & 0 & -E_1E_2S^2 & \frac{1}{2}E_1E_2CS_2 & 0 & \frac{1}{2}E_2^2CS_2 & 0 & 0 \\
E_1E_2C^2S & \frac{1}{4}E_1E_2S_2^2 & 0 & E_1E_2S^3 & 0 & \frac{1}{4}E_1E_2S_2^2 & E_2^2C^3-1 & 0 & 0 & -\frac{1}{2}E_2^2SS_2 & \frac{1}{4}E_1^2S_2\,(1+C_2) & \frac{1}{2}E_2S_2 & \frac{1}{4}E_1E_2S_2\,(C_2-1) & 0 & 0 \\
0 & 0 & 0 & 0 & -E_2S^2 & 0 & 0 & E_2^2C-1 & 0 & 0 & 0 & 0 & 0 & 0 & \frac{1}{2}E_2^2S_2 \\
0 & 0 & E_2^2S^2 & 0 & 0 & 0 & 0 & 0 & E_2^2C-1 & 0 & 0 & 0 & 0 & \frac{1}{2}E_2^2S & 0 \\
E_1E_2S^3 & -\frac{1}{4}E_1E_2S_2^2 & 0 & \frac{1}{2}E_1E_2CS_2 & 0 & -\frac{1}{4}E_2^2S_2^2 & -\frac{1}{2}E_2SS_2 & 0 & 0 & E_2^2C^3-1 & \frac{1}{4}E_1^2S_2\,(C_2-1) & -\frac{1}{2}S_2 & \frac{1}{4}E_1E_2S_2\,(1-C_2) & 0 & 0 \\
-E_1E_2CS_2 & \frac{1}{2}E_1E_2C^2S_2 & 0 & \frac{1}{2}E_1E_2SS_2 & 0 & E_1E_2C^3S & \frac{1}{2}E_2^2CS_2 & 0 & 0 & -\frac{1}{2}E_2^2CS_2 & \frac{1}{2}E_1^2C\,(1+C_2)-1 & -E_2S^2 & \frac{1}{2}E_1E_2C^2\,(C_2-1) & 0 & 0 \\
-E_1E_2CS^2 & -\frac{1}{2}E_1E_2S^2S_2 & 0 & E_1E_2CS^2 & 0 & -\frac{1}{2}E_1E_2S^2S_2 & \frac{1}{2}E_2CS_2 & 0 & 0 & -\frac{1}{2}E_2^2CS_2 & -\frac{1}{2}E_2^2S^2\,(1+C_2) & -1+\frac{1}{2}E_2^2C^2 & \frac{1}{2}E_1E_2S^2\,(1-C_2) & 0 & 0 \\
0 & \frac{1}{2}E_2^2S_2 & 0 & 0 & 0 & \frac{1}{2}E_2^2S_2 & 0 & 0 & 0 & 0 & \frac{1}{2}E_1E_2\,(C_2-1) & 0 & \frac{1}{2}E_2^2\,(1+C_2)-1 & 0 & 0 \\
0 & 0 & -\frac{1}{2}E_2^2S_2 & 0 & 0 & 0 & 0 & 0 & -E_2^2S & 0 & 0 & 0 & 0 & -1+E_2^2C^2 & 0 \\
0 & 0 & 0 & 0 & -\frac{1}{2}E_2^2S_2 & 0 & 0 & E_2^2S & 0 & 0 & 0 & 0 & 0 & 0 & -1+E_2^2C^2
\end{pmatrix}
$$

$$
\times \begin{pmatrix} a_{Iz}^{(1)} \\ a_{Ix}^{(1)} \\ a_{Iy}^{(1)} \\ a_{Sz}^{(1)} \\ a_{Sx}^{(1)} \\ a_{Sy}^{(1)} \\ a_{xz}^{(1)} \\ a_{yz}^{(1)} \\ a_{zx}^{(1)} \\ a_{zy}^{(1)} \\ a_{zz}^{(1)} \\ a_{xy}^{(1)} \\ a_{yx}^{(1)} \\ a_{xx}^{(1)} \\ a_{yy}^{(1)} \end{pmatrix}
=
\begin{pmatrix} (1-E_1)(1+E_1C)C \\ (1-E_1)(1+E_1C)S \\ 0 \\ (1-E_1)(1+E_1C)C \\ 0 \\ (E_1-1)(1+E_1C)S \\ (1-E_1)E_2S \\ 0 \\ 0 \\ (1-E_1)E_2S \\ 0 \\ 0 \\ 0 \\ 0 \\ 0 \end{pmatrix}
$$

Here trigonometric functions are abbreviated as $C = \cos\theta$, $C_2 = \cos 2\theta$, $S = \sin\theta$ and $S_2 = \sin 2\theta$. Solving the system of linear equations, gives the steady-state density operator $\hat{\rho}_1(\theta)$. The Cartesian product operator amplitudes with non-zero solutions are given by:

$$a_{Iz}^{(1)} = \frac{(E_1 - 1)\left(E_2^2 + \cos\theta\right)}{E_1 E_2^2 - 1 + \left(E_1 - E_2^2\right)\cos\theta} \tag{S72}$$

$$a_{Ix}^{(1)} = \frac{(E_1 - 1)\sin\theta}{E_1 E_2^2 - 1 + \left(E_1 - E_2^2\right)\cos\theta} \tag{S73}$$

$$a_{Sz}^{(1)} = \frac{(E_1 - 1)\left(E_2^2 + \cos\theta\right)}{E_1 E_2^2 - 1 + \left(E_1 - E_2^2\right)\cos\theta} \tag{S74}$$

$$a_{Sy}^{(1)} = \frac{(1 - E_1)\sin\theta}{E_1 E_2^2 - 1 + \left(E_1 - E_2^2\right)\cos\theta} \tag{S75}$$

$$a_{xz}^{(1)} = \frac{(E_1 - 1)\, E_2 \sin\theta}{E_1 E_2^2 - 1 + \left(E_1 - E_2^2\right)\cos\theta} \tag{S76}$$

$$a_{zy}^{(1)} = \frac{(E_1 - 1)\, E_2 \sin\theta}{E_1 E_2^2 - 1 + \left(E_1 - E_2^2\right)\cos\theta} \tag{S77}$$

The above equations can be further simplified when $\tau_{\mathrm{R}} \ll T_2 \leq T_1$, which is usually valid for the interpulse delays chosen in practice. Hence the exponential functions in Eqs. S72-S77 are expanded as a Taylor series and truncated to first order. Thus, coefficients of the density operator at start of the first (odd) evolution period ($\hat{\rho}_1$) are expressed as:

$$a_{Iz}^{(1)} = a_{Sz}^{(1)} = \frac{1 + \cos\theta}{1 + \cos\theta + 2\left(1 - \cos\theta\right)\frac{T_1}{T_2}} \tag{S78}$$

$$a_{Ix}^{(1)} = a_{xz}^{(1)} = -a_{Sy}^{(1)} = a_{zy}^{(1)} = \frac{\sin\theta}{1 + \cos\theta + 2\left(1 - \cos\theta\right)\frac{T_1}{T_2}} \tag{S79}$$

Notice that Eq. S79 is almost identical to the relation describing transverse magnetization as a function of pulse flip angle in conventional SSFP.[3,4] In analogy to conventional SSFP and within the framework of our model, the ISSFP signal also depends only on the T_1/T_2 ratio. Furthermore, Eq. S79 indicates that for each spin only one of the two observable transitions contributes to the spectrum. This result is more apparent by inspecting the terms in the

fictitious spin-1/2 basis:

$$a_{14z}^{(1)} = \frac{2\,(1+\cos\theta)}{1+\cos\theta+2\,(1-\cos\theta)\,\frac{T_1}{T_2}} \tag{S80}$$

$$a_{13x}^{(1)} = -a_{34y}^{(1)} = \frac{2\sin\theta}{1+\cos\theta+2\,(1-\cos\theta)\,\frac{T_1}{T_2}} \tag{S81}$$

For a complete understanding of the sequence we proceed by calculating the density operator after the second set of pulses ($\hat{\rho}_3$). For evaluation of $\hat{\rho}_3$ we use $\hat{\rho}_1$ as a starting point. The coefficients of the density operator in the fictitious spin-1/2 basis are given by:

$$a_{14z}^{(3)} = \frac{2\,(1+\cos\theta)}{1+\cos\theta+2\,(1-\cos\theta)\,\frac{T_1}{T_2}} \tag{S82}$$

$$-a_{24y}^{(3)} = a_{12x}^{(3)} = \frac{2\sin\theta}{1+\cos\theta+2\,(1-\cos\theta)\,\frac{T_1}{T_2}} \tag{S83}$$

The density operator $\hat{\rho}_3$ contains operators $\hat{I}_z^{(14)}$, $\hat{I}_y^{(24)}$ and $\hat{I}_x^{(12)}$, hence during the second ISSFP block the signal from the other transition for each spin is observed. This is schematically represented in the main text, Fig. 2b.

Next we analytically evaluate the ISSFP signal response to the repetition time τ_{R}. For simplicity the pulse flip angle is chosen to be $\pi/2$. Furthermore, we define an accrued phase $\Phi = \pi J \tau_{\mathrm{R}}$. The derivation is carried out in the same manner as for the flip angle dependence described above, and leads to:

$$a_{Iz}^{(1)} = \frac{(E_1-1)\,E_2\,(E_2^3+\cos 2\Phi)}{1+E_1E_2^5+E_2^2\,(E_1+E_2)\cos 2\Phi} \tag{S84}$$

$$a_{Ix}^{(1)} = \frac{(1-E_1)\,(1+E_2^3\cos 2\Phi)}{1+E_1E_2^5+E_2^2\,(E_1+E_2)\cos 2\Phi} \tag{S85}$$

$$a_{Iy}^{(1)} = \frac{(E_1-1)\,E_2\,(1+E_2^3)\cos\Phi}{1+E_1E_2^5+E_2^2\,(E_1+E_2)\cos 2\Phi} \tag{S86}$$

$$a_{Sz}^{(1)} = \frac{(E_1-1)\,E_2\,(E_2^3+\cos 2\Phi)}{1+E_1E_2^5+E_2^2\,(E_1+E_2)\cos 2\Phi} \tag{S87}$$

$$a_{Sx}^{(1)} = \frac{(E_1-1)\,E_2\,(1+E_2^3)\cos\Phi}{1+E_1E_2^5+E_2^2\,(E_1+E_2)\cos 2\Phi} \tag{S88}$$

$$a_{Sy}^{(1)} = \frac{(1-E_1)\left(1+E_2^3\cos 2\Phi\right)}{1+E_1E_2^5+E_2^2\left(E_1+E_2\right)\cos 2\Phi} \tag{S89}$$

$$a_{xz}^{(1)} = \frac{(E_1-1)\,E_2\left(E_2^3-1\right)\sin\Phi}{1+E_1E_2^5+E_2^2\left(E_1+E_2\right)\cos 2\Phi} \tag{S90}$$

$$a_{yz}^{(1)} = \frac{(E_1-1)\,E_2^3\sin 2\Phi}{1+E_1E_2^5+E_2^2\left(E_1+E_2\right)\cos 2\Phi} \tag{S91}$$

$$a_{zx}^{(1)} = \frac{(E_1-1)\,E_2^3\sin 2\Phi}{1+E_1E_2^5+E_2^2\left(E_1+E_2\right)\cos 2\Phi} \tag{S92}$$

$$a_{zy}^{(1)} = \frac{(E_1-1)\,E_2\left(E_2^3-1\right)\sin\Phi}{1+E_1E_2^5+E_2^2\left(E_1+E_2\right)\cos 2\Phi} \tag{S93}$$

$$a_{xx}^{(1)} = \frac{(1-E_1)\,E_2^2\sin 2\Phi}{1+E_1E_2^5+E_2^2\left(E_1+E_2\right)\cos 2\Phi} \tag{S94}$$

$$a_{yy}^{(1)} = \frac{(E_1-1)\,E_2^2\sin 2\Phi}{1+E_1E_2^5+E_2^2\left(E_1+E_2\right)\cos 2\Phi} \tag{S95}$$

The density operator dependence on the repetition time τ_R is now more complex and several additional terms, including double-quantum terms, play a role in the spin dynamics. Notice that when $\tau_\mathrm{R} = \frac{k}{2J}$, where k is an odd integer, terms with $\cos\Phi$ and $\sin 2\Phi$ in the numerator vanish and maximal transverse magnetization is achieved. Additionally, at these maxima the density operator contains $\hat{I}_x^{(13)}$ and $\hat{I}_y^{(34)}$ for $k = 1, 5, 9\ldots$, while operators $\hat{I}_y^{(24)}$ and $\hat{I}_x^{(12)}$ are present for $k = 3, 7, 11\ldots$, which indicates that the observed transitions for each spin alternate between the maxima.

Analysis of the ISSFP$_\text{xy-x-y}$ sequence

Here we examine the ISSFP$_\text{xy-x-y}$ sequence analytically following the same approach as descried above. However, twice as many steps are required as the sequence is comprised of four blocks. As a result of the additional complexity for the derivation we also assume that $T_1 = T_2$. However, even with this assumption solutions of the density operator coefficients could only be obtained for describing the τ_R dependence. All nonzero solutions are then

given by:

$$a_{Iz}^{(1)} = \frac{E^2}{1+E+E^2} \tag{S96}$$

$$a_{Ix}^{(1)} = \frac{-E\cos\Phi}{1+E+E^2} \tag{S97}$$

$$a_{Iy}^{(1)} = -\frac{1}{1+E+E^2} \tag{S98}$$

$$a_{Sz}^{(1)} = \frac{E^2}{1+E+E^2} \tag{S99}$$

$$a_{Sx}^{(1)} = \frac{-E\cos\Phi}{1+E+E^2} \tag{S100}$$

$$a_{Sy}^{(1)} = -\frac{1}{1+E+E^2} \tag{S101}$$

$$a_{yz}^{(1)} = \frac{E\sin\Phi}{1+E+E^2} \tag{S102}$$

$$a_{zy}^{(1)} = \frac{E\sin\Phi}{1+E+E^2} \tag{S103}$$

Here E represents $\exp[-\tau_R/T]$ with $T = T_1 = T_2$. Notice that even for small accrued phases Φ, a substantial amount of transverse magnetization is generated. This indicates that polarization transfer occur with high efficiency for systems with small J-coupling using very short repetition times $\tau_R \ll \frac{1}{J}$.

Materials and Methods

Samples

In the present study D_2O (Cortecnet) solutions of 1 M sodium formate (natural abundance, Sigma-Aldrich), 20 mM D-glucose-^{13}C-1 (Cortecnet) and 2 M sodium lactate (natural abundance, Sigma-Aldrich) were investigated.

NMR spectroscopy

All NMR experiments were performed on an 11.7 T magnet interfaced to a Bruker Advance Neo console equipped with a TCI Prodigy probe. The spectrometer operates at Larmor frequencies of 498.83 MHz and 125.43 MHz for ^{1}H and ^{13}C, respectively. The ^{1}H and ^{13}C chemical shifts were referenced to a ^{1}H signal of H_2O at 4.70 ppm. Unless stated otherwise all experiments utilized pulses with a radiofrequency (RF) amplitude of 25 kHz for both ^{1}H and ^{13}C.

All ^{13}C steady-state experiments were acquired for a duration $> 5T_1^{\mathrm{C}}$, typically for ca. $10T_1^{\mathrm{C}}$. A 20 μs dead time τ_{de} was included prior to acquisition τ_{acq}. The specific repetition time $\tau_{\mathrm{R}} = \tau_{\mathrm{de}} + \tau_{\mathrm{acq}}$ and the pulse flip angle θ for each steady-state experiment, are provided in Figure 1 and S2-S5. The spectra were processed using in-house written scripts in MATLAB. To avoid potential artifacts only time domain data after $5T_1$ were used in the processing. The free induction decay (FID) from each block were co-added, Fourier transformed (FT) and phase corrected. The resulting spectra were then added together for SSFP and $\mathrm{ISSFP}_{\mathrm{xy\text{-}x\text{-}y}}$ sequences. 0.5 kHz continuous wave (CW) ^{1}H RF was used in the decoupled/NOE SSFP sequence.

^{13}C INEPT experiments were acquired using the refocused INEPT sequence with or without ^{1}H decoupling. The experiment recycle delay was $1.3T_1^H$ and all interpulse delays were set to $1/4J$, where the value of the ^{1}H-^{13}C J-coupling constant was determined experimentally for each system. 2 kHz CW ^{1}H decoupling RF was used for the sodium formate and D-glucose samples, while 4 kHz decoupling using garp4[5] was employed for sodium lactate. The time domain data was zero-filled, multiplied by an exponential weighting function and Fourier transformed. The line-broadening of the weighting function was chosen to maximize the signal-to-noise ratio (SNR) of the peak in the spectrum.

The SNR per square root unit time ($\mathrm{SNR_t}$) was computed from the maximum peak intensity of the real part of the spectrum and standard deviation of noise obtained from a separate experiment, which had same the processing and experimental parameters except

all pulse powers were set to 0 W. More specifically, the $\mathrm{SNR_t}$ for the steady-state sequences was calculated according to the following equation:

$$\mathrm{SNR_t} = \frac{\max\left(\mathrm{real}\left(\mathrm{signal}\right)\right)}{\sigma\left(\mathrm{real}\left(\mathrm{noise}\right)\right)\sqrt{b \cdot N \cdot \tau_{\mathrm{R}}}}, \tag{S104}$$

where $\max\left(\mathrm{real}\left(\mathrm{signal}\right)\right)$ is the maximum peak intensity in the real part of the spectrum, $\sigma\left(\mathrm{real}\left(\mathrm{noise}\right)\right)$ is the standard deviation of the real part of the noise spectrum, b is the number of blocks in the steady-state sequence and N is the number of loops over which the signal was co-added. As explained in the main text the spectra from the two blocks in the $\mathrm{ISSFP_{xy}}$ sequence were not added, hence the $\mathrm{SNR_t}$ was calculated for each block separately according to Equation S104. The total $\mathrm{SNR_t}$ was then evaluated by summing the maximum signal intensity of both blocks:

$$\mathrm{SNR_t} = \frac{\max\left(\mathrm{real}\left(\mathrm{signal_1}\right)\right) + \max\left(\mathrm{real}\left(\mathrm{signal_2}\right)\right)}{\sigma\left(\mathrm{real}\left(\mathrm{noise}\right)\right)\sqrt{b \cdot N \cdot \tau_{\mathrm{R}}}}, \tag{S105}$$

where $\max\left(\mathrm{real}\left(\mathrm{signal_1}\right)\right)$ and $\max\left(\mathrm{real}\left(\mathrm{signal_2}\right)\right)$ are the maximum peak intensity in the real part of the spectrum from first and second block, respectively. $\sigma\left(\mathrm{real}\left(\mathrm{noise}\right)\right)$ is the standard deviation of the real part of the co-added noise spectrum. For INEPT experiments the $\mathrm{SNR_t}$ was calculated analogously according to:

$$\mathrm{SNR_t} = \frac{\max\left(\mathrm{real}\left(\mathrm{signal}\right)\right)}{\sigma\left(\mathrm{real}\left(\mathrm{noise}\right)\right)\sqrt{\mathrm{ns} \cdot \left(d_1 + \tau_{\mathrm{acq}}\right)}}, \tag{S106}$$

where d_1 is the recycle delay and ns the number of scans. The error of $\mathrm{SNR_t}$ was estimated by repeating the experiments in quadruplicate.

MRI

Phantom tests were done on 5 mm NMR tubes with 50 mM D-glucose-^{13}C1 dissolved in a 2% H_2O agarose phantom, on a 15.2 T Bruker horizontal imaging scanner running Paravision

6. Acquisitions used a double-resonance homebuilt surface coil tuned to 649.93 (^{1}H) and 167.7 MHz (^{13}C) frequencies. Figure S2 shows the SSFP, decoupled SSFP, and SSFP$_{\text{xy-x-y}}$ sequences used. Additional imaging parameters included matrix size of 32x32, FOV of 40x40 mm^2, acquisition time of 1.2 ms (12 points), 10 kHz acquisition bandwidth, repetition time $\tau_{\text{R}} = 2.41$ ms, and weighted average along the phase-encoding axes according to

$$\#scans(n) = (\text{int}) \left\{ 1.5 + (\text{NA} - 1) \cdot 0.5 \cdot \left[1 + \cos\left(\frac{4\pi n}{N} \right) \right] \right\}, \tag{S107}$$

where N = NA = 32. The ^{13}C offset was set in all cases on-resonance on the α-form glucose-C1 peak (96.6 ppm); the ^{1}H offset for the decoupled experiment was set at 4.7 ppm, while for the ISSFP$_{\text{xy-x-y}}$ it was set at 4.5 ppm. In the latter sequence, simultaneous slice selective pulses of duration 0.315 ms were applied on the ^{13}C and ^{1}H channels. ^{1}H irradiation in the decoupled/NOE images used 0.2W and an mlev16[6] modulation.

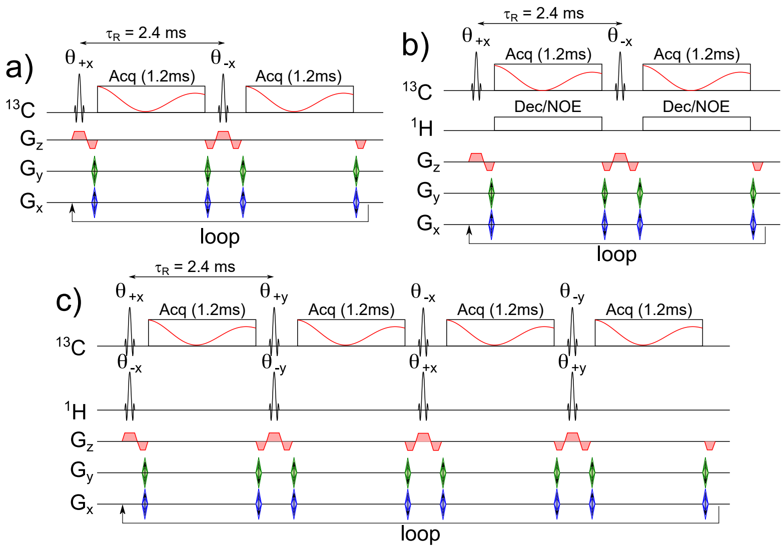

Figure S2: ^{13}C MRI balanced SSFP pulse sequences assayed in this work and leading to the results in Figure 3. (a) Conventional 2D SSFP. (b) SSFP with decoupling/NOE. (c) ISSFP$_{xy-x-y}$. The slice-selective pulses were tuned to select an axial cut of the agarose phantom, while x/y gradients imaged the phantom in-plane. In all cases, τ_{R} refers to the spacing between consecutive pulses. Data was processed by Fourier transforming the short FIDs, as well as the k_x and k_y phase encodings. See text for additional acquisition parameters.

Simulations

All numerical simulations were performed using SpinDynamica 3.8.0 in Mathematica 13.0.[7] Relaxation in the simulations is included phenomenologically as implemented in Spindynamica. Unless stated otherwise, the relaxation time constants in the simulations for all the terms in the density operator were 1 s. All pulses are assumed to be ideal.

Experimental and simulation results

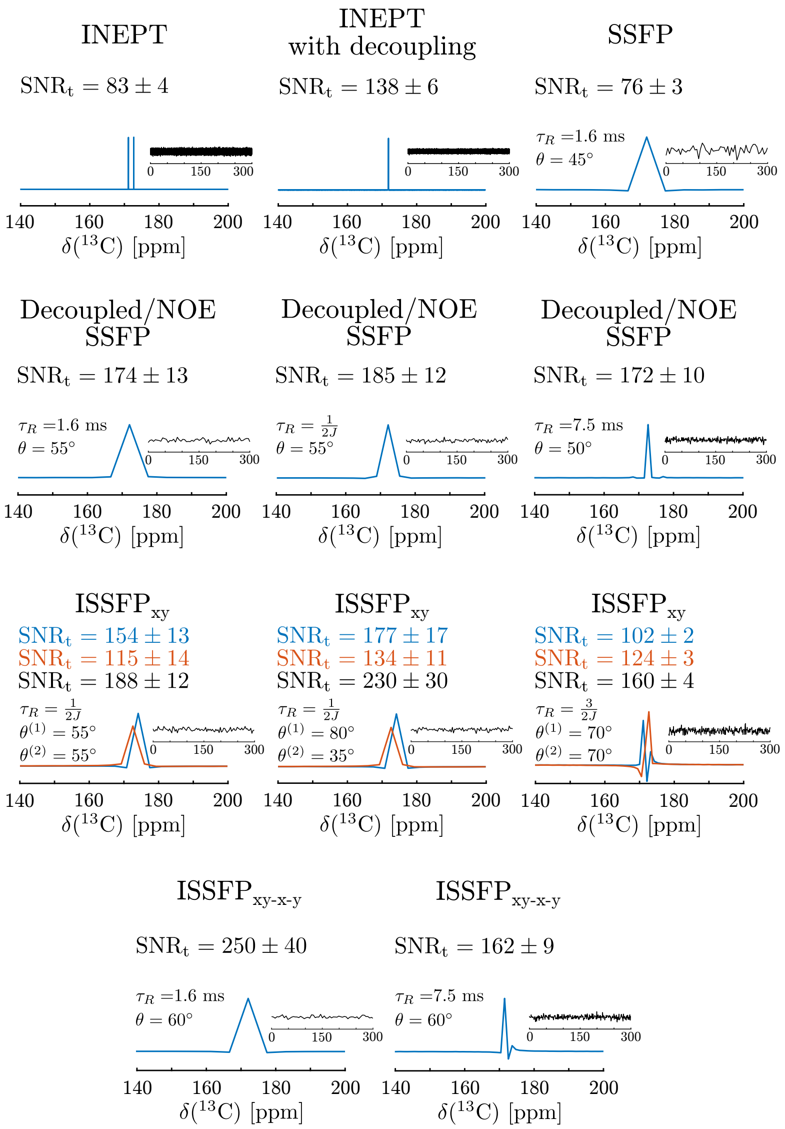

Figure S3: ^{13}C NMR spectra of 1 M sodium formate solution in D_2O acquired using different pulse sequences: INEPT, INEPT with ^1H decoupling, SSFP, SSFP with ^1H irradiation (decoupled/NOE SSFP), ISSFP$_{xy}$ and ISSFP$_{xy\text{-}x\text{-}y}$. For each spectrum an inset with a separate (100× magnification) noise spectrum is given, acquired under the same conditions, but without pulsing. The red and blue spectra from ISSFP$_{xy}$ represent the spectrum from the first (odd) and second (even) block, while the shown noise inset is of the first (odd) block. For the steady-state experiments the chosen repetition time τ_R and flip angle θ are shown. In the ISSFP$_{xy}$ sequence ^{13}C and ^1H flip angles are represented by $\theta^{(1)}$ and $\theta^{(2)}$, respectively, while in the ISSFP$_{xy\text{-}x\text{-}y}$ sequence flip angles θ for both nuclei are equal. Additionally, the calculated SNR$_t$ for each spectrum is provided.

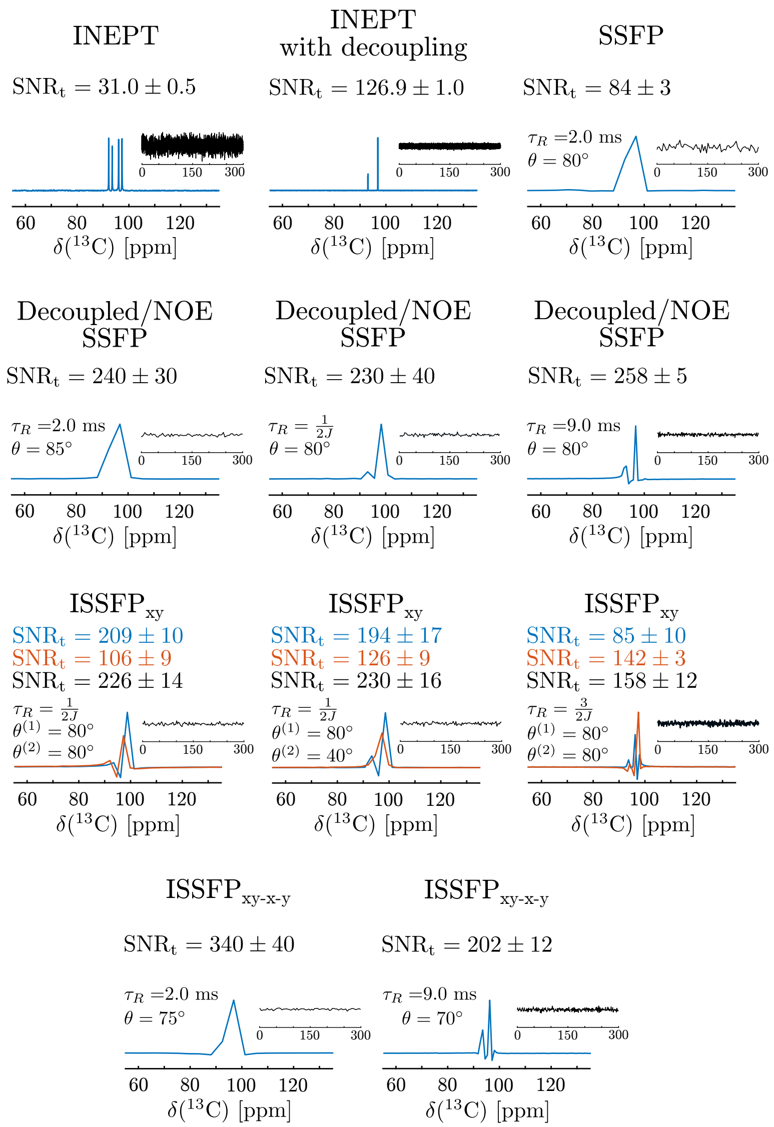

Figure S4: ^{13}C NMR spectra of a 20 mM D-glucose-^{13}C-1 solution in D_2O acquired using different pulse sequences: INEPT, INEPT with ^1H decoupling, SSFP, SSFP with ^1H irradiation (decoupled/NOE SSFP), ISSFP$_{xy}$ and ISSFP$_{xy\text{-}x\text{-}y}$. For each spectrum an inset with a separate (50× magnification) noise spectrum is given, acquired under the same conditions, but without pulsing. The red and blue spectra from ISSFP$_{xy}$ represent the spectrum from the first (odd) and second (even) block, while the shown noise inset is of the first (odd) block. For the steady-state experiments the chosen repetition time τ_R and flip angle θ are shown. In the ISSFP$_{xy}$ sequence ^{13}C and ^1H flip angles are represented by $\theta^{(1)}$ and $\theta^{(2)}$, respectively, while in the ISSFP$_{xy\text{-}x\text{-}y}$ sequence flip angles θ for both nuclei are equal. Additionally, the calculated SNR$_t$ for each spectrum is provided. When spectral resolution is sufficiently high, the two forms (α and β) of glucose appear resolved. For all experiments, the carrier frequency was placed on-resonance with the α-form.

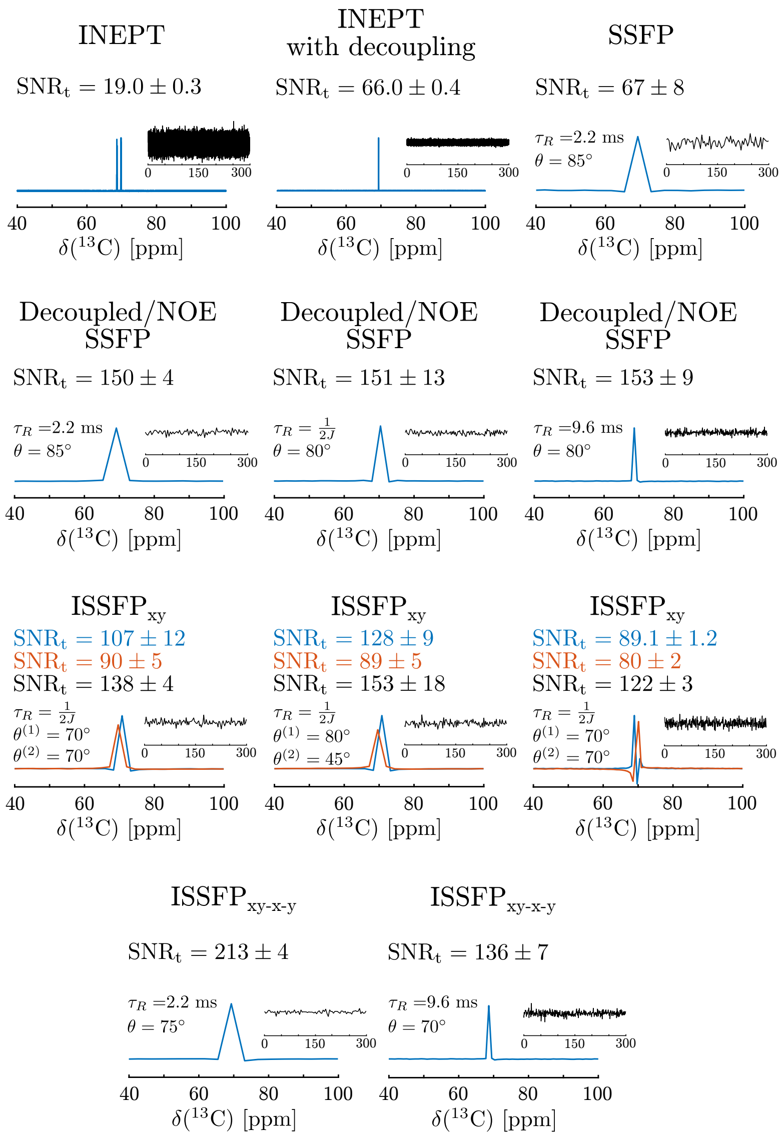

Figure S5: ^{13}C NMR spectra of 2 M sodium lactate solution in D_2O acquired using different pulse sequences: INEPT, INEPT with ^{1}H decoupling, SSFP, SSFP with ^{1}H decoupling, ISSFP$_{xy}$ and ISSFP$_{xy\text{-}x\text{-}y}$. The absolute value of the ISSFP$_{xy}$ spectra are shown. For each spectrum an inset with a separate (50× magnification) noise spectrum is given, acquired under the same conditions, but without pulsing. The red and blue spectra from ISSFP$_{xy}$ represent the spectrum from the first (odd) and second (even) block, while the shown noise inset is of the first (odd) block. For the steady-state experiments the chosen repetition time τ_R and flip angle θ are shown. In the ISSFP$_{xy}$ sequence ^{13}C and ^{1}H flip angles are represented by $\theta^{(1)}$ and $\theta^{(2)}$, respectively, while in the ISSFP$_{xy\text{-}x\text{-}y}$ sequence flip angles θ for both nuclei are equal. Additionally, the calculated SNR$_t$ for each spectrum is provided.

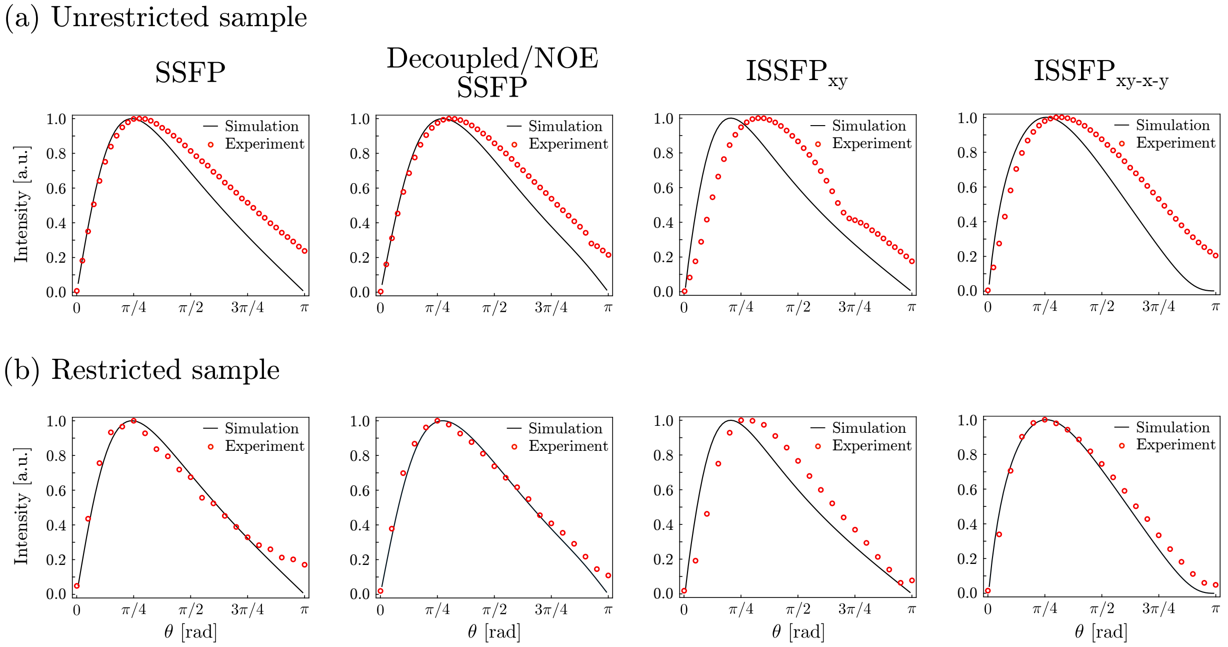

Figure S6: Simulated and experimental steady-state signal intensity of ^{13}C in a sodium formate D_2O solution as a function of flip angle θ. (a) shows the signal dependence on the flip angle from sample in a 5 mm NMR tube. (b) shows the obtained signal dependence on the flip angle from sample in a 3 mm Shigemi tube. The repetition times used in the sequences were 2.0 ms. The simulations were performed using the experimentally measured relaxation constants of sodium formate given in Table S1.

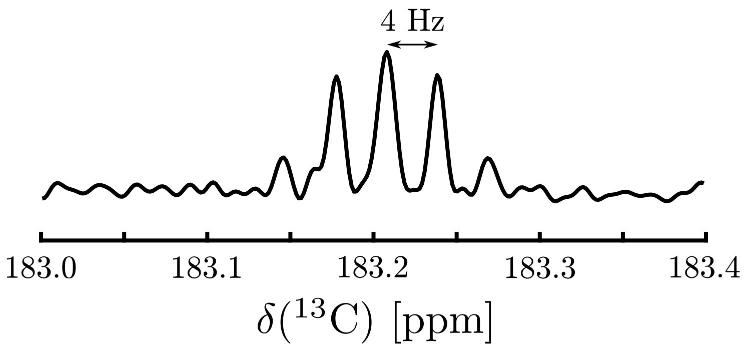

Figure S7: ^{13}C spectrum of lactate's carboxyl moiety for a 2 M sodium lactate solution in D_2O. The spectrum was acquired using a single pulse experiment with 4 scans and a recycle delay of 10 s.

Table S1: ^{1}H and ^{13}C relaxation time constants

Sample		δ [ppm]	T_1 [s]	T_2 [s]
Sodium formate	^{1}H	8.51	13.5 ± 0.5	3.90 ± 0.09
	^{13}C	172.0	19.9 ± 0.8	4.2 ± 0.2
Glucose	^{1}H	4.67	1.30 ± 0.05	1.21 ± 0.04
	^{13}C	96.7	1.85 ± 0.10	0.85 ± 0.06
Sodium lactate	^{1}H	4.13	3.1 ± 0.2	2.9 ± 0.2
	^{13}C	69.2	4.95 ± 0.10	3.6 ± 0.6
	^{13}C	183.2	27 ± 3	3.0 ± 0.9

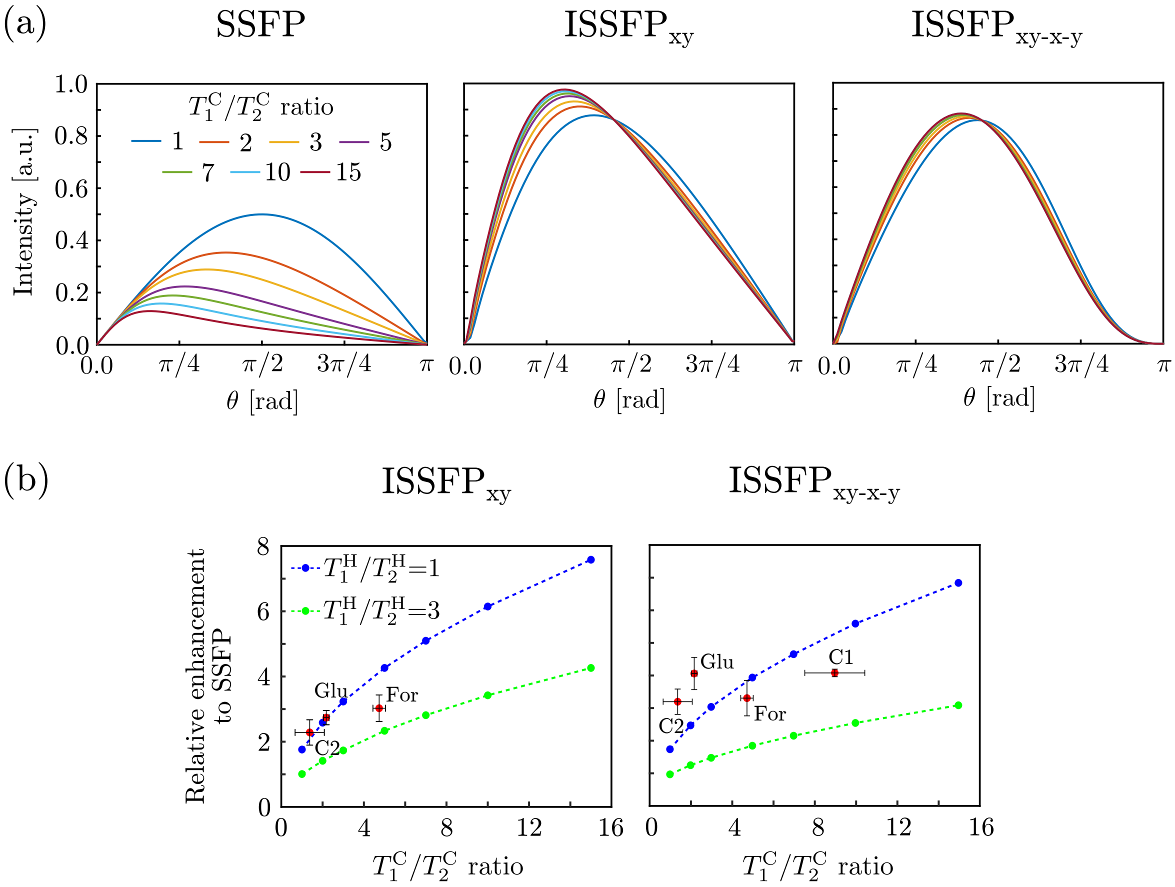

Figure S8: (a) Simulated steady-state ^{13}C signal intensity dependence as a function of flip angle θ for different T_1^C/T_2^C ratios. (b) Relative enhancements of ISSFP$_{xy}$ and ISSFP$_{xy\text{-}x\text{-}y}$ sequences with respect to SSFP. Red circles mark the experimentally observed enhancements for ^{13}C in sodium formate (For), D-glucose-^{13}C-1 (Glu) and sodium lactate C1 and C2 carbon sites. In the simulations the chosen repetition times were 0.1 ms and $1/(2J)$=2.5 ms for the SSFP and ISSFP sequences, respectively.

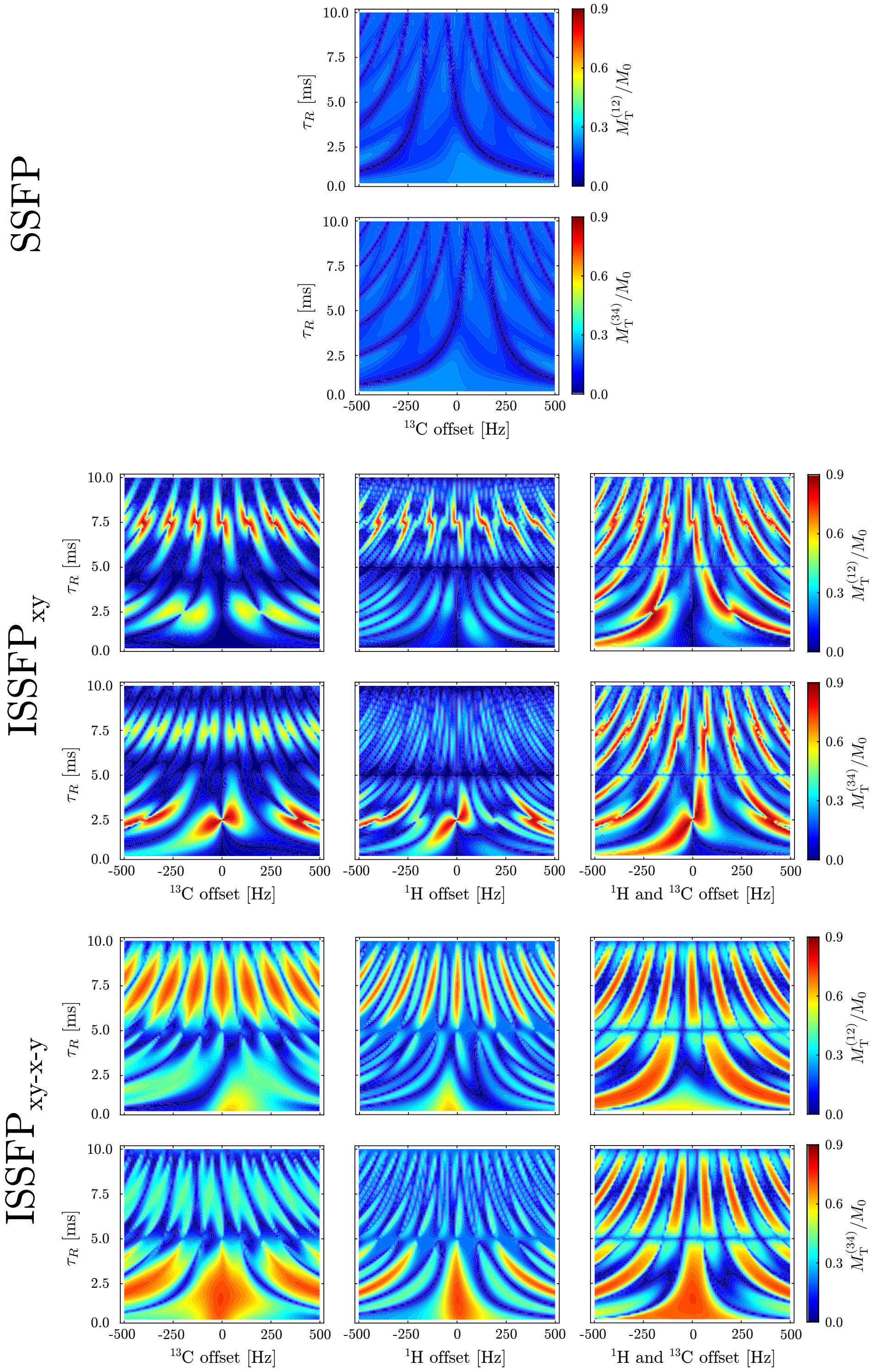

Figure S9: Simulations of steady-state signal intensity dependence on offset. From top to bottom, simulations show the steady-state ^{13}C transverse magnetization at the beginning of the first (odd) block for the SSFP, ISSFP$_{xy}$ and ISSFP$_{xy\text{-}x\text{-}y}$ sequences as a function of repetition time τ_R and ^{13}C, ^{1}H or ^{13}C and ^{1}H offset. Separate plots are provided for each of the two observable ^{13}C transitions ($1 \rightarrow 2$ and $3 \rightarrow 4$). In the simulations the chosen flip angle for all sequences was $\pi/2$.

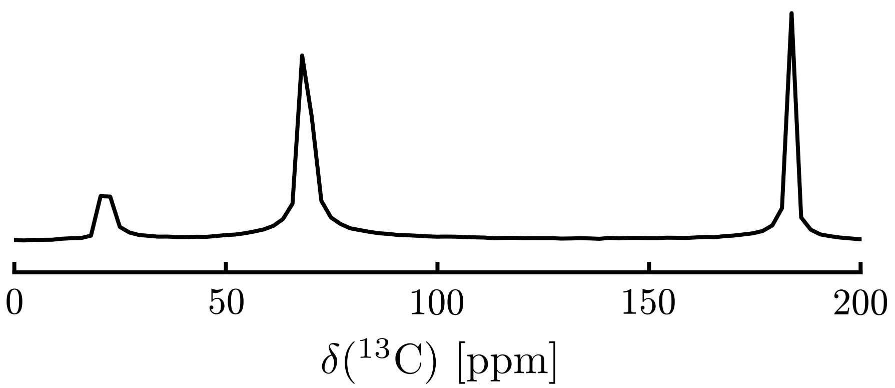

Figure S10: ^{13}C spectrum of a 2 M sodium lactate solution in D_2O acquired using $\mathrm{ISSFP_{xy\text{-}x\text{-}y}}$ sequence. The repetition time τ_R was 3.6 ms and flip angle θ was 65°. The carrier frequency for ^{13}C was set on resonance with lactate's carboxyl moiety at 183.2 ppm, and ^{1}H was on resonance with proton of the methine group at 4.13 ppm.

References

(1) Sørensen, O.; Eich, G.; Levitt, M. H.; Bodenhausen, G.; Ernst, R. Product operator formalism for the description of NMR pulse experiments. *Prog. Nucl. Magn. Reson. Spectrosc.* **1984**, *16*, 163–192.

(2) Vega, S. Fictitious spin 1/2 operator formalism for multiple quantum NMR. *J. Chem. Phys.* **1978**, *68*, 5518–5527.

(3) Hinshaw, W. S. Image formation by nuclear magnetic resonance: The sensitive-point method. *J. Appl. Phys.* **1976**, *47*, 3709–3721.

(4) Schwenk, A. Steady-state techniques for low sensitivity and slowly relaxing nuclei. *Prog. Nucl. Magn. Reson. Spectrosc.* **1985**, *17*, 69–140.

(5) Shaka, A.; Barker, P. B.; Freeman, R. Computer-optimized decoupling scheme for wide-band applications and low-level operation. *J. Magn. Reson.* **1985**, *64*, 547–552.

(6) Levitt, M. H.; Freeman, R.; Frenkiel, T. Broadband heteronuclear decoupling. *Journal of Magnetic Resonance (1969)* **1982**, *47*, 328–330.

(7) Bengs, C.; Levitt, M. H. SpinDynamica: Symbolic and numerical magnetic resonance in a Mathematica environment. *Magn. Reson. Chem.* **2018**, *56*, 374–414.
